# Portable geosmin detection system based on sensor cells expressing insect odorant receptors

**DOI:** 10.1038/s41598-026-41786-8

**Published:** 2026-04-28

**Authors:** Hidefumi Mitsuno, Shogo Araki, Yuji Sukekawa, Daigo Terutsuki, Sawako Niki, Eri Kuroda, Shunsuke Fujibayashi, Takeshi Sakurai, Kumiko Oguma, Satoshi Yamaguchi, Shinya Yamahira, Teruyuki Nagamune, Ryohei Kanzaki

**Affiliations:** 1 Research Center for Advanced Science and Technology, The University of Tokyo, 4-6-1 Komaba, Meguro-ku, Tokyo, 153-8904 Japan; 2 Graduate School of Information Science and Technology, The University of Tokyo, 7-3-1 Hongo, Bunkyo-ku, Tokyo, 113-8656 Japan; 3 Department of Agricultural Innovation for Sustainability, Faculty of Agriculture, Tokyo University of Agriculture, 1737 Funako, Atsugi-shi, 243-0034 Kanagawa Japan; 4 Department of Urban Engineering, Graduate School of Engineering, The University of Tokyo, 7-3-1 Hongo, Bunkyo-ku, Tokyo, 113- 8656 Japan; 5 Department of Chemistry and Biotechnology, The University of Tokyo, 7-3-1 Hongo, Bunkyo-ku, Tokyo, 113-8656 Japan; 6 Present Address: Department of Mechanical Engineering and Robotics, Faculty of Textile Science and Technology, Shinshu University, 3-15-1, Tokida, Ueda City, Nagano, 386-8567 Japan; 7 Present Address: SANKEN, The University of Osaka, 8-1, Mihogaoka, 567-0047 Ibaraki, Osaka Japan

**Keywords:** Portable measurement, Odor sensor, Bio-sensing, Insect odorant receptor, Sf21 cell line, Geosmin, Biomaterials - cells, Lab-on-a-chip

## Abstract

A technology that enables highly sensitive, selective on-site detection of odorants without sophisticated equipment could be valuable for a range of applications, from water quality testing to disease diagnosis. In this study, we introduce a portable technology using Sf21 cells engineered to express *Drosophila melanogaster* odorant receptors, co-receptors, and calcium-sensitive fluorescent proteins for geosmin detection—a natural compound known for its distinct musty odor. Sensor cartridges were fabricated by immobilizing sensor cells on the glass surface inside tubes. When coupled with a portable fluorometer, the system successfully detected geosmin at concentrations as low as 100 pM (18.2 ppt) without requiring any concentration steps. Moreover, the system was deployed at a lakeside dam and detected geosmin in real field samples. This is the first successful detection of target odorants using biosensors comprising transfected sensor cells bearing specific odorant receptor genes. The results highlight the practicality of insect odorant receptor-expressing sensor cells and reveal the potential of this technology in various applications, such as food, beverage, and water quality testing, where on-site odorant detection is required.

**Supplementary Information:**

The online version contains supplementary material available at 10.1038/s41598-026-41786-8.

## Introduction

Moldy odors are a widespread issue impacting the safety and acceptability of drinking water^1^. Providing safe and suitable water is critical for preventing waterborne diseases and ensuring global well-being and prosperity^2^. Drinking water sources, including rivers, lakes, dam reservoirs, and wells, are vulnerable to excessive blooms of planktonic algae, cyanobacteria, and actinomycetes, which may produce moldy odors^3,4^. Consequently, many water treatment facilities routinely monitor moldy odorant concentrations at water intakes. For example, Japanese drinking water standards stipulate that moldy odorants, such as 2-methyl-isoborneol (2-MIB) and geosmin, must not exceed 10 ng/L (10 parts per trillion; ppt). If this threshold is surpassed, advanced treatment methods—such as oxidation and activated carbon adsorption—are implemented^5–9^.

Current detection methods for moldy odors in water treatment plants include human sensory evaluation and the more sophisticated gas chromatography–mass spectrometry (GC/MS). To enhance sensitivity for detecting low concentrations (ppt level) of key moldy odorants like geosmin, GC/MS is often combined with solid-phase microextraction (SPME) or purge-and-trap techniques, which concentrate geosmin for precise analysis^10,11^. However, GC/MS requires large, specialized equipment and skilled personnel in dedicated laboratories, increasing the time and cost required; in addition, water quality may be altered during transportation. Moreover, human sensory evaluation is inherently subjective and cannot reliably quantify geosmin due to olfactory fatigue, adaptation, and inter-individual variation in sensory perception. This necessitates a simple technology for sensitive, on-site detection of moldy odors.

In recent decades, portable odor sensors have been introduced for chemical detection. For example, breathalyzers for alcohol monitoring and electronic noses (E-Noses) designed for chemical recognition have become common^12,13^. Despite these advances, current sensors face limitations regarding sensitivity, selectivity, and application range, with most being restricted to the gas phase^14–16^. To overcome these challenges, various detection technologies based on the olfactory system of living organisms have been proposed, ranging from those using living organisms, such as dogs, mice, and insects, to those using biological molecules, such as odorant receptors (ORs), antibodies, enzymes, and DNA aptamers^17–26^.

Insects possess numerous olfactory receptor neurons (ORNs) in their antennae, which utilize odorant receptors and olfactory receptor co-receptor (Orco) to detect trace amounts of odorants^27,28^. Diverse ORs in *Drosophila melanogaster* and *Anopheles gambiae* have been identified, including those specific for pheromones and geosmin, and others responsive to a wide range of odorants^29–31^. Distinct from mammalian ORs (G-protein coupled receptors), insect ORs function as ligand-gated ion channels that form complexes with Orco, opening in response to odorants^32–35^. Upon odorant binding, these ORs undergo conformational changes that non-selectively allow extracellular cations into the cell, facilitating affinity measurements via ionic current analysis.

Based on this principle, sensor technologies exploiting insect ORs have been developed^36–45^. Previously, a compact fluidic chip using *Xenopus laevis* oocytes expressing insect ORs and Orco served as an odorant sensor^36^. Furthermore, sensor cells co-expressing insect ORs, Orco, and the calcium-sensitive fluorescent protein GCaMP have been used as elements that display fluorescent changes in response to target components^38^. Cell-array chips containing four sensor cells on glass substrates can detect target chemicals by distinct fluorescent patterns and respond to gaseous odorants^39,45^. Thus, these cells have been employed as odorant sensor components. Nevertheless, such measurements require laboratory-based equipment, including electrophysiological devices and fluorescence microscopes, with no suitable technology available for field deployment.

**Fig. 1:**
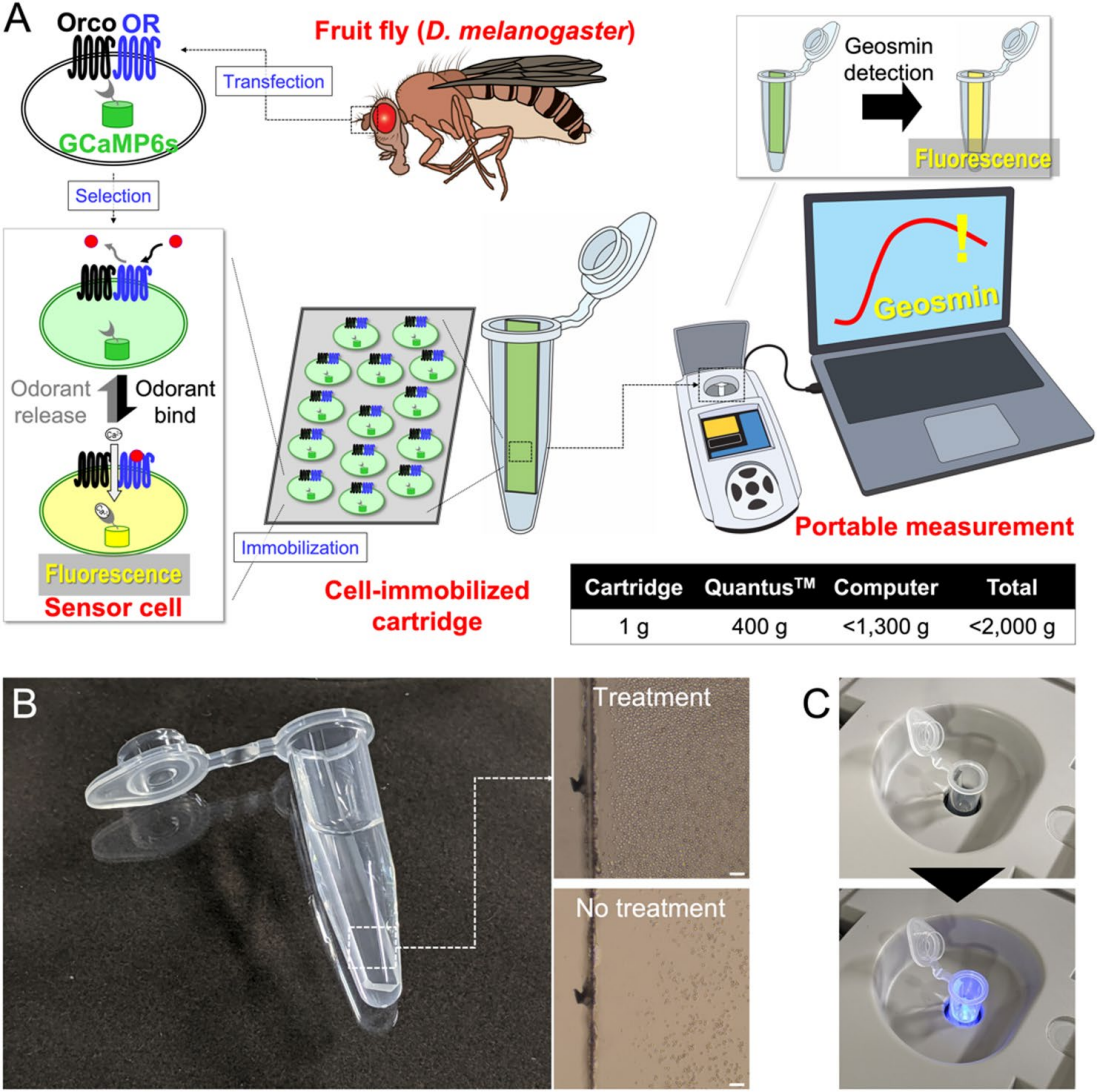
Schematic illustration of insect odorant receptor-based on-site geosmin sensing technology. **(A)** Target odorant receptor (OR) and *Orco* genes are isolated from the antennae of target insect species, *Drosophila melanogaster*. By introducing three genes (*OR*, *Orco*, and *GCaMP6s*) into Sf21 cells, we generated cells with high fluorescence response to the target odorant. Next, we isolated single cells and established a cell line with excellent odorant responsiveness as sensor cells. The established sensor cells were immobilized on a glass substrate with a cell-immobilizing agent and combined with a tube to construct a cell-immobilized cartridge. The cell-immobilized cartridge can be set in a fluorometer, such as Quantus™, to detect the target odorant in the sample based on the change in the fluorescence intensity of the sensor cell. The table shows the weight of each component; the standard weight of the computer used in this study is shown as an example. **(B)** Photograph of a cell-immobilized cartridge and transparent images of the glass surfaces. The glass surface of the cell immobilization agent-treated glass (Treatment) and no-treated glass (No-treatment) are shown. Scale bar = 100 μm. **(C)** Photograph of a cartridge inserted into Quantus™ and during measurement.

In this study, we sought to develop a portable technology for detecting target odorants. Odor-sensing Sf21 cells were engineered to co-express the geosmin-specific insect OR, Orco, and calcium-sensitive fluorescent protein GCaMP6s, enabling reliable detection of target odorants via fluorescence intensity changes. Following performance evaluations—including dose–response and odorant selectivity analyses—sensor cells were established. Additionally, cartridges for sensor cell immobilization were fabricated and integrated with a portable fluorometer to measure fluorescence responses, resulting in a portable system for field detection of geosmin (Fig. 1).

## Results

### Selection of target odorant receptors (ORs)

We targeted geosmin and 2-methylisoborneol (2-MIB) as specific moldy-odorant components of drinking water, alongside 1-octen-3-ol as a moldy-odorant control. To develop sensors that specifically detect these moldy odorants, we consulted the *Drosophila* Database of Odorant Responses (DoOR, http://neuro.uni-konstanz.de/DoOR/default.html)^46,47^. Our initial search focused on ORs for geosmin, revealing that ab4B neurons exhibited the strongest response, with an olfactory response measured as a model response of 0.573. Or56a, which is expressed in ab4B neurons, has been identified as sensitive to geosmin^48^ and was thus, selected for use in this study.

Similarly, we used DoOR to identify ORs responsive to 2-MIB and observed that Or19a showed the highest model response (0.351) among the ORs. Nevertheless, Or19a showed greater responses to valencene (model response = 0.775) and additional odorants than to 2-MIB, suggesting that *D. melanogaster* lacks a specific OR for 2-MIB. Consequently, we did not explore the detection of 2-MIB further in this study. Finally, we searched for ORs sensitive to 1-octen-3-ol and found that Or13a yielded a strong model response (0.789), leading us to select it as the target OR.

### Establishment of OR-expressing sensor cells

To develop a practical sensor, we generated cell lines that produce a robust and consistent fluorescent response to geosmin. We accomplished this by transfecting Sf21 cells with either the wild-type (WT) *Or56a* gene or a codon-optimized *Or56a* gene of *D. melanogaster* (tailored for Sf21 cells), along with the *Orco* and *GCaMP6s* genes. This process yielded two distinct cell lines—Or56a-WT and Or56a-SF. As fluorescent intensity depended on transgene expression levels and varied among individual cells, we selected 11 Or56a-WT and 19 Or56a-SF clones exhibiting the highest transgene expression using the limiting dilution and cell proliferation methods (Supplementary Fig. S1, Table S1). We then screened these cell lines for changes in fluorescence after exposure to 10 µM geosmin.

**Fig. 2:**
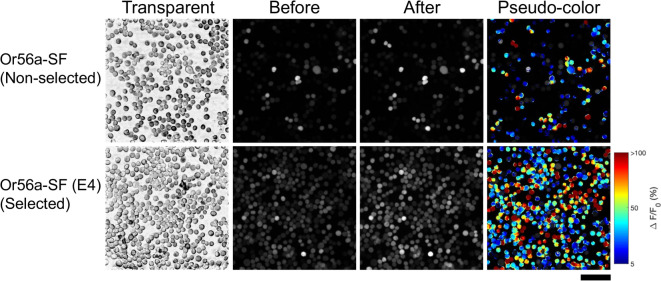
Fluorescence response of cells to geosmin. Microscope images of typical fluorescence changes in a group of non-selected cells transfected with the *Or56a* gene (Or56a-SF) and Or56a sensor cells (Or56a-SF (E4)) are shown. The left sides show bright-field images, the middle shows fluorescence microscopic images before and after geosmin addition, and the right sides show pseudo-color images with fluorescence responses to geosmin. The images show pseudo-color, with dark red colors indicating high fluorescence intensity change and dark blue colors indicating low fluorescence intensity change. Scale bar = 100 μm.

Most Or56a-SF and Or56a-WT clones demonstrated minimal fluorescent responses to geosmin (< 5%), but four clones from each group had increases exceeding 15% (Supplementary Table S1, Table S2). Among these, Or56a-SF-E4 and Or56a-WT-F8 showed the strongest fluorescence response (> 30%) in calcium imaging and were thus, selected as sensor cells (Fig. 2, Supplementary Fig. S2A, B).

Compared with unselected populations, these sensor cells showed greater fluorescence shifts in response to geosmin. To evaluate improvements in responsiveness, we compared the distribution and magnitude of response values between non-selected cell lines and the Or56a-WT-F8 and Or56a-SF-E4 clones (Fig. 2, Supplementary Fig. S2A). In non-selected cells, response values for both WT and codon-converted receptors were evenly spread across zones, indicating a mixture of low- and high-responders (Supplementary Fig. S2B). In contrast, the Or56a-WT-F8 and Or56a-SF-E4 sensor cells’ response values were concentrated on the high-response side, indicating a more homogenous population with high responsiveness (Supplementary Fig. S2C). This suggests that the selected sensor cells were homogeneous and exhibited stronger fluorescence responses to geosmin (Supplementary Fig. S2D).

Similarly, to generate Or13a sensor cells, we transfected Sf21 cells with *Or13a*, *Orco*, and *GCaMP6s* and isolated single cells (Supplementary Tables S1 and S3). Four cell lines were obtained, and one clone (Or13a-WT-C4) was selected for its maximal response (Supplementary Fig. S3).

RT-PCR analysis revealed that Or56a-SF-E4 sensor cells expressed all three relevant genes (*Or56a*, *Orco*, and *GCaMP6s*; Supplementary Fig. S4). Similarly, the Or13a-WT-C4 sensor cells expressed *Or13a*, *Orco*, and *GCaMP6s*. Thus, the sensor cell responses stemmed from the introduced genes. Hence, Or56a-SF-E4 and Or13a-WT-C4 were selected for subsequent experiments.

### Odorant selectivity and dose dependency of sensor cells

**Fig. 3:**
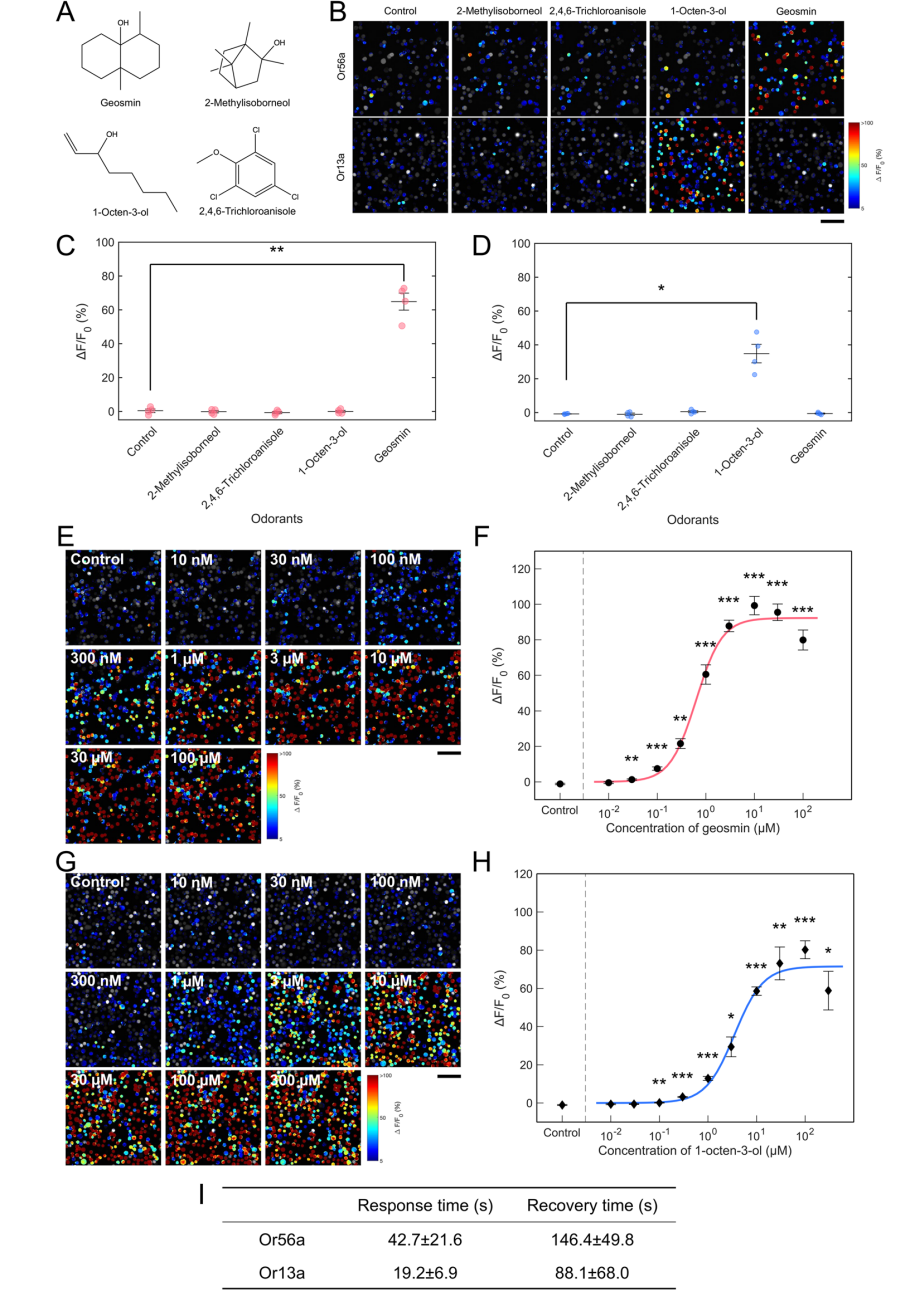
Odorant selectivity and dose-dependent responses in Or56a or Or13a sensor cells. **(A)** Chemical structures of tested moldy odorants. **(B)** Microscope images of typical fluorescence changes to tested moldy odorants in Or56a-SF-E4 sensor cells and Or13a-WT-C4 sensor cells. Each odorant was added at a concentration of 10 µM. Moldy odorant selectivity in Or56a-SF-E4 sensor cells **(C)** or Or13a-WT-C4 sensor cells **(D)**. Error bars indicate standard error (± SEM). Four experiments were performed for odorant selectivity experiments. Significant differences in fluorescence intensities against control were determined using Welch’s *t*-test with the Holm–Bonferroni correction. *p < 0.05, **p < 0.01. Microscope images of typical fluorescence changes to the indicated concentrations of geosmin in Or56a-SF-E4 sensor cells **(E)** and the dose-dependent curve **(F)**. Microscope images of typical fluorescence changes to the indicated concentrations of 1-octen-3-ol in Or13a-WT-C4 sensor cells **(G)** and the dose-dependent curve **(H)**. Error bars indicate standard error (± SEM). Six experiments were performed for dose-dependent response experiments. Significant differences in fluorescence intensities against control were determined using Welch’s *t*-test with the Holm–Bonferroni correction. *p < 0.05, **p < 0.01, ***p < 0.001. Scale bar = 100 μm. **(I)** Means of response time and recovery time of each cell in Or56a-SF-E4 and Or13a-WT-C4 sensor cells. For Or56a or Or13a, the response time and recovery time were calculated from the fluorescence responses to 1 µM of geosmin or 3 µM of 1-octen-3-ol in **(F)** or **(H)**, respectively. Data include standard error (± SD; *n* = 230 cells in Or56a-SF-E4, *n* = 339 cells in Or13a-WT-C4).

We assessed the odorant selectivity and dose-dependent responses of the Or56a-SF-E4 and Or13a-WT-C4 sensor cells toward the mold-related odorants found in tap water—geosmin and 2-MIB—as well as other odorants, such as 1-octen-3-ol and 2,4,6-trichloroanisole (Fig. 3A). The Or56a-SF-E4 sensor cells exhibited increased fluorescence intensity upon exposure to geosmin, but showed no response to 2-MIB, 2,4,6-trichloroanisole, or 1-octen-3-ol (Fig. 3B, C, and Supplementary Fig. S5A). Conversely, the control Or13a-WT-C4 sensor cells displayed a strong fluorescence response to 1-octen-3-ol without responding to geosmin, 2-MIB, or 2,4,6-trichloroanisole (Fig. 3B, D, and Supplementary Fig. S5A). These results demonstrate that the established sensor cells can selectively detect specific components among the tested mold-related odorants based on the intrinsic specificity of the expressed receptors.

Additionally, Or56a-SF-E4 sensor cells demonstrated a dose-dependent increase in fluorescence ranging from 30 nM to 10 µM, with detectable limits beginning at 30 nM (Fig. 3E, F). Similarly, Or13a-WT-C4 sensor cells showed a dose-dependent fluorescence response within the 100 nM–100 µM range, and a detection threshold of 100 nM (Fig. 3G, H). Both sensor cells exhibited a fluorescent response to each odorant, followed by a return to baseline and heightened fluorescence when exposed to incrementally higher concentrations (Fig. 3E, G, and Supplementary Fig. S5B). Based on the dose–response curves, the half-maximal effective concentration (EC_50_) for Or56a and Or13a sensor cells was 0.63 µM for geosmin and 3.50 µM for 1-octen-3-ol (Fig. 3F, H).

Response and recovery times were determined by monitoring changes in individual cell fluorescence over time following odorant exposure. The Or56a-SF-E4 and Or13a-WT-C4 sensor cells exhibited response times of 42.7 ± 21.6 s and 19.2 ± 6.9 s, with corresponding recovery times of 146.4 ± 49.8 and 88.1 ± 68.0 s, respectively (Fig. 3I). Although the stimulus duration was consistent at 15 s across experiments (see Methods), Or56a-SF-E4 sensor cells generally presented longer response and recovery times compared to Or13a-WT-C4 cells, likely attributable to differences in the chemical and physical properties of the respective odorants (Supplementary Fig. S6).

Collectively, these results suggest that both the type and concentration of mold-related odorants can be discerned through variations in sensor cell fluorescence intensity. Accordingly, the efficacy of Or56a-SF-E4 as a geosmin sensor was evaluated using real-world samples.

### Detection of geosmin in surface water

Specificity is an essential characteristic of sensors, enabling the accurate detection of target molecules without interference from non-target odorants (i.e., background odors) and contaminants. To assess the specificity of the fluorescence response of Or56a-SF-E4 sensor cells to geosmin, samples were collected from a Dam Reservoir in Japan—a source of drinking water that may contain mold-related odorants, as well as various other odorants and contaminants resulting from living organisms and anthropogenic pollution, typical of many natural surface waters.

**Fig. 4:**
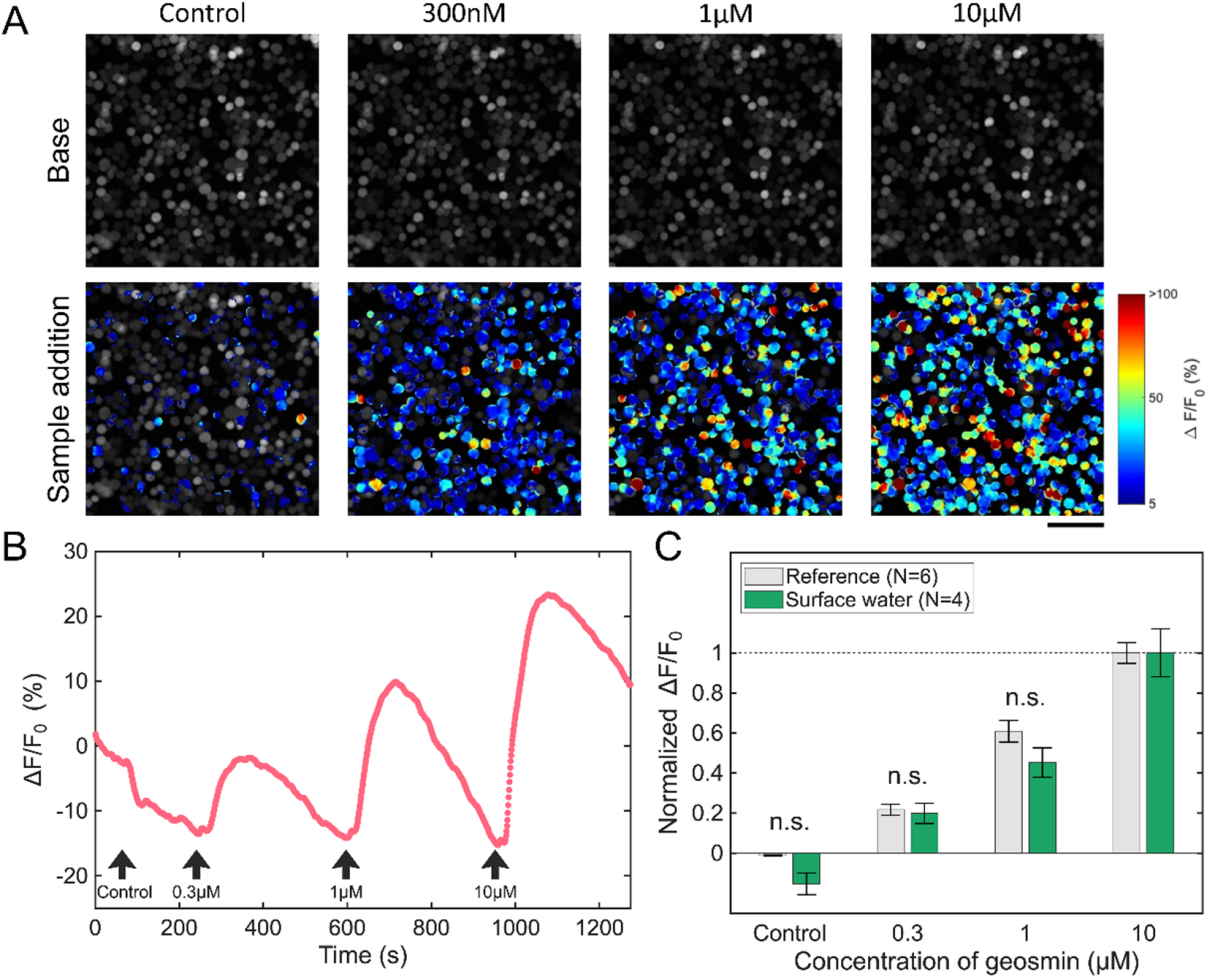
Geosmin detection by Or56a-SF-E4 sensor cells in surface water sample. **(A)** Fluorescence images of Or56a-SF-E4 sensor cells in response to surface water samples with and without geosmin (Control, 300 nM, 1 µM, and 10 µM). The images were acquired under a fluorescence microscope and subsequently used for analysis. Fluorescence intensities before and after sample addition are represented by pseudo-color. Scale bar = 100 μm. **(B)** Trace of measurement of surface water samples. Black arrows show the times when surface water samples were added in the order: Control, 300 nM, 1 µM, and 10 µM. **(C)** Fluorescence intensity change for geosmin samples prepared with ultrapure water (Reference sample) and surface water. The responses were normalized based on the response to 10 µM geosmin. Error bars indicate the standard error of the mean (± SEM, *n* = 4 for surface water samples, *n* = 6 for reference samples). A Welch’s *t*-test with the Holm–Bonferroni correction was used for the reference and surface water samples at the same concentration. There were no significant differences (n.s. = not significant at 5% significance level).

To determine if sensor cell fluorescence corresponds with geosmin concentration in real-world (surface water) samples, measurements were conducted at three levels (0.3, 1, and 10 µM) that displayed linear responses per the concentration–response curve in Fig. 3F. A marked increase in fluorescence intensity was recorded from Or56a-SF-E4 sensor cells exposed to surface water supplemented with various geosmin concentrations (0.3, 1, and 10 µM), compared with the negative control sample (Control), which exhibited no fluorescence signal (Fig. 4A).

Consistent with results in ultrapure water, fluorescence intensity in surface water peaked following sample addition and subsequently returned to baseline values (Fig. 4B). The changes in fluorescence intensity also increased with increasing geosmin concentration.

Subsequently, dose–response curves were compared between samples prepared with ultrapure water and those from the reservoir’s surface water. First, to compare with the standard dose–response curve in Fig. 3F, the fluorescence intensity at each point (control, 0.3 µM, and 1 µM) was normalized to the value measured at 10 µM. Control samples yielded intensity readings of −0.01 ± 0.00 (ultrapure water) and − 0.15 ± 0.05 (surface water). In contrast, geosmin-containing samples presented values of 0.22 ± 0.02 (0.3 µM) and 0.61 ± 0.05 (1 µM) for ultrapure water, while surface water samples showed 0.20 ± 0.05 (0.3 µM) and 0.45 ± 0.07 (1 µM; Fig. 4C). No significant differences in fluorescent signals or responsiveness to geosmin were observed between ultrapure and surface water samples, suggesting that Or56a-SF-E4 sensor cells reliably detect geosmin regardless of the presence of contaminants.

### Geosmin detection using a sensor cell-immobilized cartridge

When developing a portable detection system, two critical factors must be addressed: (1) reducing measurement variability and (2) designing a compact device capable of recording and displaying changes in fluorescence intensity from immobilized sensor cells. To this end, we immobilized Or56a-SF-E4 sensor cells on cartridges, employed a portable fluorometer, and assessed odorant selectivity, dose-dependent responses, and the system’s suitability for on-site measurements.

**Fig. 5:**
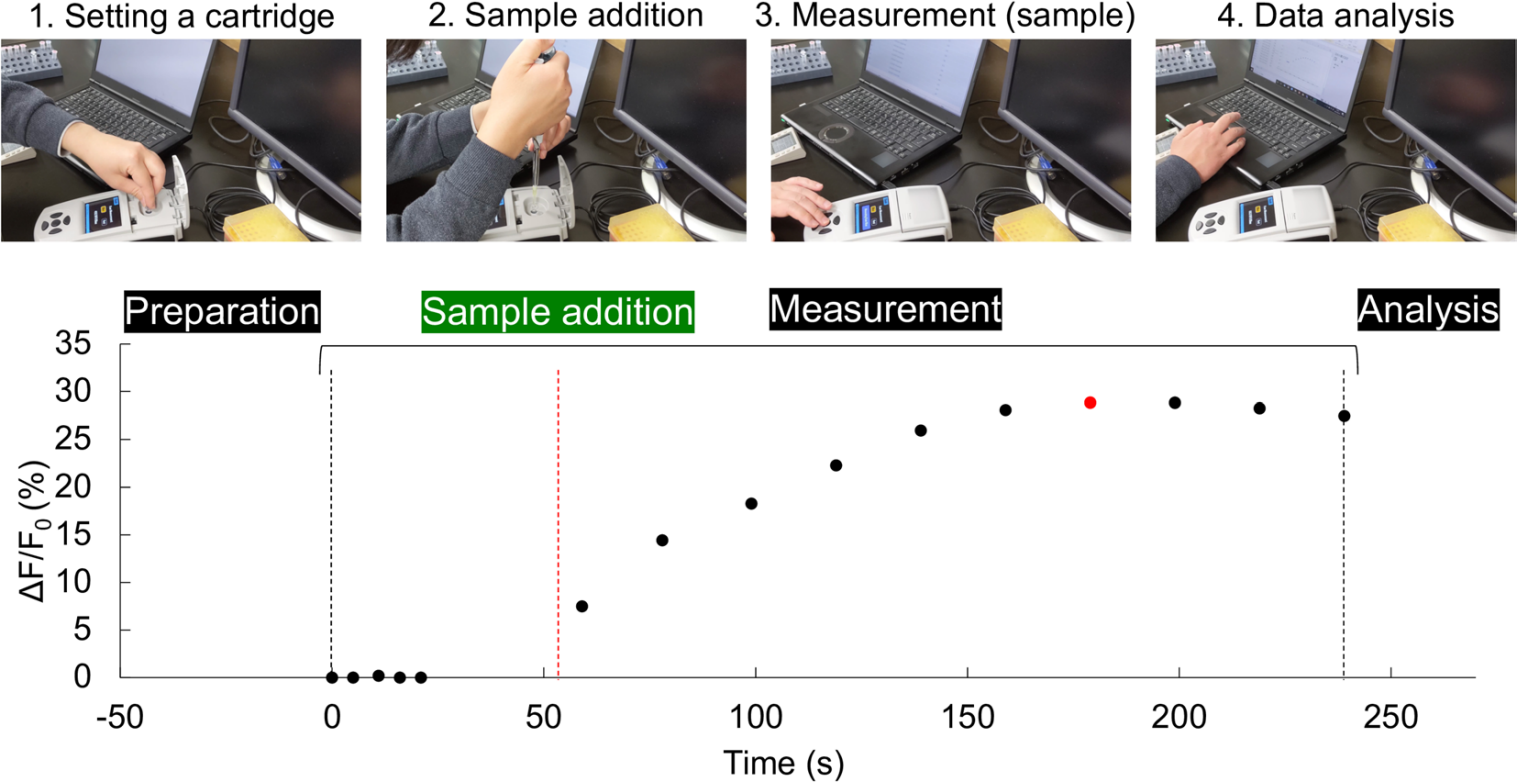
Measurement using a sensor cell-immobilized cartridge. The operational flow of the portable measurement is shown along with the actual measured data. A movie of this operation is provided in the Supplementary Materials (Movie S1). (1) Setting a cartridge: The sensor cell-immobilized cartridge is set to the fluorometer, Quantus™, and five consecutive measurements are taken, with fluorescence intensity changes at ± 2%. (2) Sample addition: After stable measurement, the sample is placed on the cartridge and pipetted a few times. (3) Measurement (sample): After closing the lid and completing the first measurement, measurements are taken every 20 s for at least 120 s. (4) Data analysis: After measurement, fluorescence intensity changes are determined each time and analyzed, and the value after approximately 120 s (red plot) is recorded as a fluorescence response. Fluorescence intensity change for a 1 µM geosmin sample is shown.

Oleyl-PEG-NHS was selected as the immobilization agent based on its proven efficacy in immobilizing various cell types^39,49^. Initially, cartridges were assembled by immobilizing sensor cells onto glass treated with oleyl-PEG-NHS, which were placed into 0.5 mL tubes. Fluorescence responses of the sensor cells were then acquired using the Quantus™ Fluorometer (Fig. 5, Supplementary Movie S1). After stabilizing fluorescence intensity variations, 200 µL of 10 µM geosmin was added to 400 µL of assay buffer in each tube. The rate of change in fluorescence intensity was measured over 140 s at 20 s intervals. This procedure was repeated with multiple sensor cell-immobilized cartridges.

For each cartridge, fluorescence intensity changes in response to geosmin were monitored, with peak intensity observed at least 120 s after geosmin addition (Fig. 5).

### Odorant selectivity using the cartridge

**Fig. 6:**
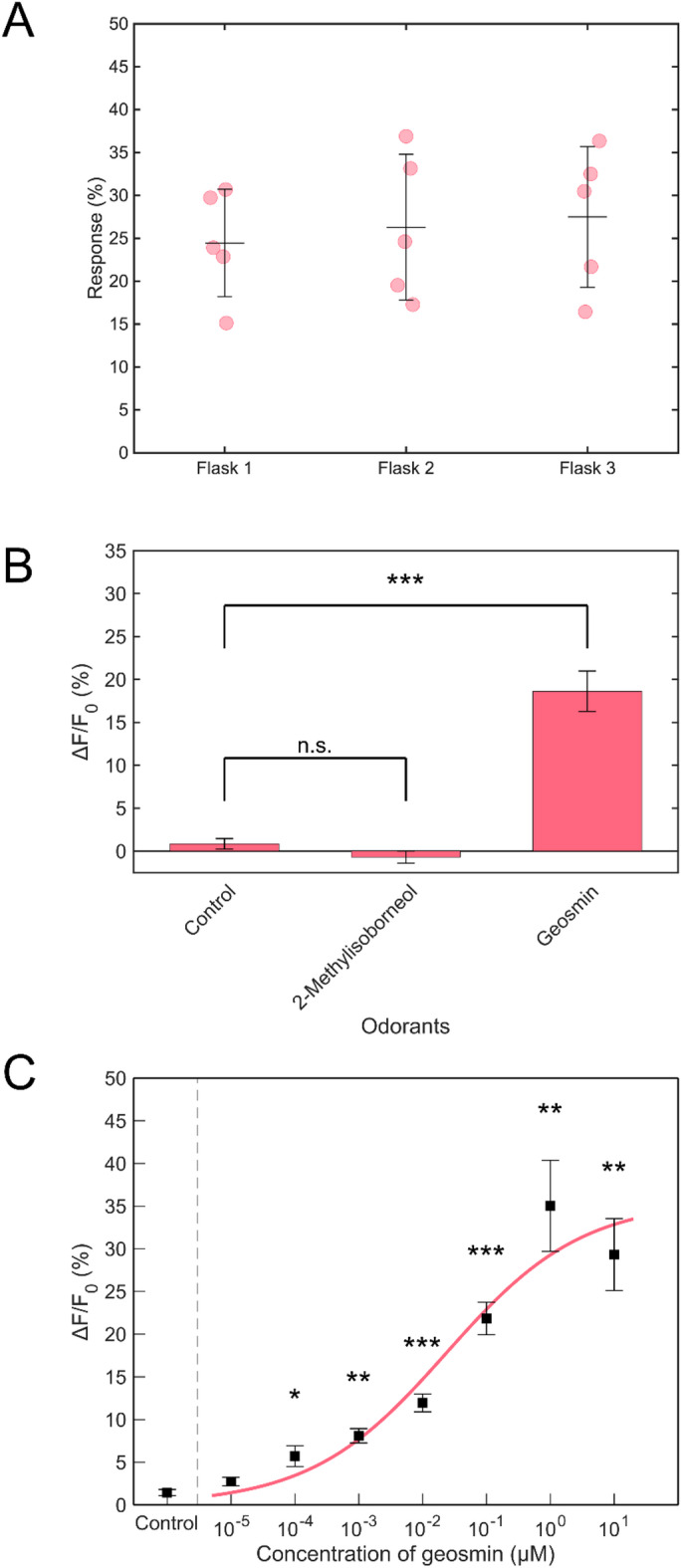
Dose-dependent fluorescence response and odorant selectivity using a sensor cell-immobilized cartridge. **(A)** Variability among cartridges. The variability in responses among cell-immobilized cartridges prepared in different flasks is shown. Cartridges were stimulated with 100 nM geosmin. Error bars are standard deviation (± SD, *n* = 5). **(B)** Selectivity of Or56a sensor cell-immobilized cartridge for moldy odorants. Geosmin and 2-MIB were added at a concentration of 10 µM; no odorants were added in the Control sample. Error bars are standard error (± SEM, *n* = 3–9). Significant differences were determined using Welch’s *t*-test with the Holm–Bonferroni correction. n.s. = not significant, ***p < 0.001. **(C)** Dose-dependent fluorescence responses of Or56a sensor cell-immobilized cartridge. Fluorescence intensity changes with respect to the concentration of geosmin from 10 pM to 100 µM are shown; no geosmin (0 M) was added to the Control samples. Error bars show standard error (± SEM, *n* = 6). Significant differences were determined using Welch’s *t*-test with the Holm–Bonferroni correction. *p < 0.05, **p < 0.01, ***p < 0.001.

Additional experiments were conducted to assess operational stability and batch-to-batch response consistency. Sensor cells cultured in separate flasks were used to prepare cell-immobilized cartridges, and their response values were compared. All sensor cells were cultured under identical conditions (1 mL of cell suspension added to 4 mL of fresh medium, cultured for 2 days) before cartridge preparation. Fluorescence changes induced by 100 nM geosmin were measured using five cartridges from each of three independent flasks. The observed fluorescence intensity changes were 24.5 ± 6.3%, 26.3 ± 8.5%, and 27.5 ± 8.2% for the respective flasks (Fig. 6A). Average response levels were comparable across flasks, with similar variability (± 6.3%–8.5%). No statistically significant differences were observed among flasks (one-way analysis of variance [ANOVA], *p* = 0.825), confirming operational stability and minimal batch-to-batch variation. These findings demonstrate that reliable sensor performance can be achieved when cell-immobilized cartridges are prepared under uniform conditions.

We also evaluated the selectivity of the Or56a sensor cell-immobilized cartridge for additional moldy odorants, such as 2-MIB. Selectivity for 10 µM of each moldy odorant was determined by measuring fluorescence intensity 120 s following compound addition. Relative to the buffer-only sample, geosmin elicited a fluorescence change of 18.6 ± 2.4% (*p* < 0.001), while 2-MIB resulted in a change of −0.7 ± 0.7% (not significant; Fig. 6B). A clear difference was also observed in the fluorescence intensity between geosmin and 2-MIB. These results indicate that the Or56a sensor cell-immobilized cartridge demonstrates specific detection of geosmin.

### Dose-dependent response of the cartridge

Fluorescence intensity was evaluated as a function of geosmin concentration. A significant increase in fluorescence was observed at geosmin concentrations exceeding 100 pM (*p* < 0.05; Fig. 6C). The dose–response curve showed that the cartridge exhibited a notable response over the 100 pM to 1 µM geosmin concentration range, with an EC_50_ of 23.7 nM. This value closely aligns with the average geosmin concentration in dams and lakes (~ 10 ng/L or 10 ppt). Accordingly, the cartridge’s detection limit was determined to be 100 pM. At this concentration, considering geosmin’s molecular weight (182.3), 100 pM equates to 18.2 ng/L, or 18.2 ppt. Conventional water quality analysis methods typically require pre-concentration of geosmin from water samples—using techniques such as purge-and-trap or SPME—prior to GC/MS analysis^5^. In contrast, the established sensor cells were capable of detecting geosmin at ppt levels without any pre-concentration steps, indicating a detection limit comparable to that achieved with GC/MS.

### On-site application of geosmin detection technology

**Fig. 7:**
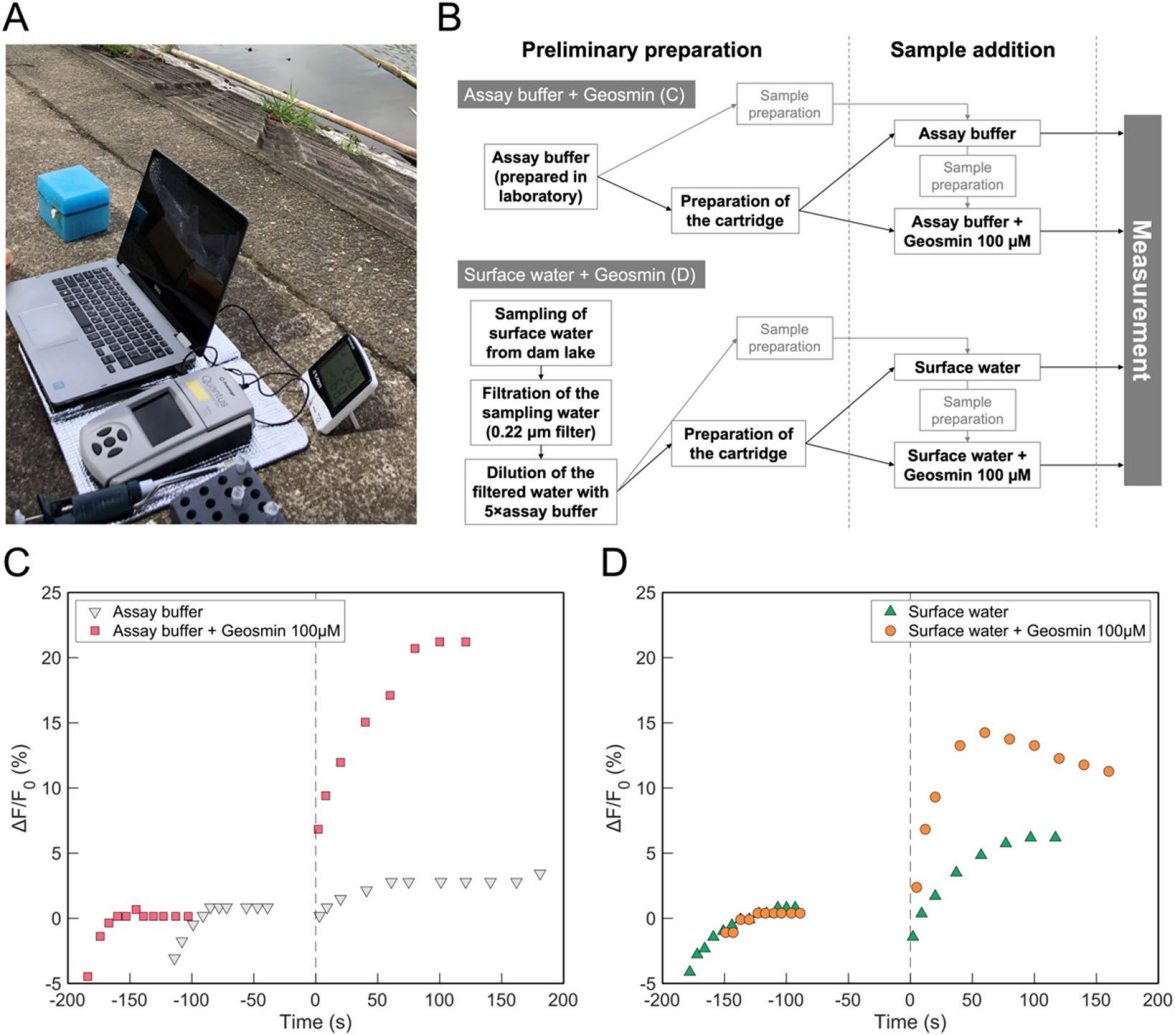
On-site detection using sensor cell-immobilized cartridge. **(A)** Photograph of the measurement at the dam lakeside of a water supply source. The cell-immobilized cartridge, Quantus™, and a laptop computer were used to test water samples collected from the lake. **(B)** Flowchart of the on-site measurements. The flowchart of the measurements of the prepared solution in the laboratory and the surface water collected in the dam lake is shown. In the former, the assay buffer was prepared with pure water in the laboratory, and the samples and cartridges were prepared with the assay buffer in the field for measurement. In the latter case, the assay buffer was prepared using filtered surface water collected in the dam lake, and the samples and cartridges were prepared in the field. The measurements were performed in the same manner, except that assay buffer or surface water buffer was used. **(C)** Traces of fluorescence intensity change over time for an assay buffer prepared from the pure water (gray triangle) and another sample prepared by mixing 100 µM geosmin into the assay buffer (red square). The sample was added at 0 s (dotted line) after more than five stable measurements within ± 2% of the fluorescence intensity change. **(D)** Traces of fluorescence intensity change over time for a sample prepared from the surface water of the dam lake (green triangle) and another sample prepared by mixing 100 µM geosmin into a surface water sample (orange circle). The sample was added at the timing of the dotted line (at 0 s) after more than five measurements within ± 2% of the fluorescence intensity change. GC-MS measurements were conducted at the Water Quality Center of the Prefectural Waterworks Bureau and confirmed that 301.7 pM (total, 0.055 µg/L; 55 ppt) geosmin was present in the dam lake sample at the time of sample collection.

We evaluated the applicability of the developed geosmin detection technology for on-site analysis using water samples collected from the Dam Reservoir. The experimental setup comprised cell-immobilization cartridges, a Quantus™ Fluorometer, and a laptop computer, all arranged at the lakeside, where fluorescence intensity measurements of the sensor cells were performed using laboratory-prepared samples (Fig. 7A, B). Consistent with the laboratory findings, time-dependent changes in fluorescence intensity were observed at 2.8% with assay buffer alone and at 21.2% with assay buffer containing 100 µM geosmin (Fig. 7C). These results confirmed that cartridge-based geosmin detection is feasible in both field and laboratory environments.

Further evaluation involved measuring geosmin in surface water collected directly from the lake using the cartridges (Fig. 7B). The change in fluorescence intensity was 6.0% for surface water alone and 13.7% for surface water supplemented with 100 µM geosmin (Fig. 7D).

According to GC/MS analyses conducted concurrently by a Prefectural Waterworks Bureau, the water sample contained approximately 104.2 pM (excluding algae) or 301.7 pM (total) geosmin (Supplementary Tables S4 and S5). Collectively, these findings demonstrate that the Or56a sensor cell-immobilized cartridge effectively detected geosmin on-site, encompassing both dam reservoir and lakeside conditions.

## Discussion

In this study, we developed a portable odorant-sensing technology designed for the field detection of target moldy odorants in water. We established homogeneous sensor cells by expressing functional insect ORs, Orco, and GCaMP6s. By isolating single cells and selecting lines with high responsiveness, we generated sensor cells that exhibit highly selective, sensitive fluorescent responses to geosmin. Furthermore, we engineered sensor cell-immobilized cartridges to facilitate straightforward, portable detection of moldy odorants in water. This demonstrated the feasibility of on-site geosmin detection using the sensor cell-immobilized cartridges.

Regarding selectivity, Or56a was found to specifically respond to geosmin. In this study, the Or56a-SF-E4 sensor cells yielded fluorescent signals only upon exposure to geosmin, without cross-reactivity to other mold-related odorants. Similarly, Or13a-WT-C4 sensor cells selectively responded to 1-octen-3-ol, consistent with previous findings^46,50^, but not to geosmin, 2-MIB, or 2,4,6-trichloroanisole. Previous reports confirm that Or13a responds to chemicals related to 1-octen-3-ol, but not to the other odorants tested in this study^39,44,51,52^. Leveraging the unique selectivity profiles of these sensor cells, we constructed an Or56a-SF-E4 sensor cell-immobilized cartridge tailored for geosmin detection, thus demonstrating the potential for target-specific odorant identification based on individual sensor cell properties.

Moreover, the sensitivity of our platform is comparable to standard analytical instrumentation (Table 1). This sensor-cell-based device has a total mass of 2 kg (Fig. 1) and enables rapid measurements, requiring only a few minutes from sample introduction to result acquisition. Thus, this technology has great potential as a compact, simple, and rapid method for on-site measurement of geosmin in water.

**Table 1:**
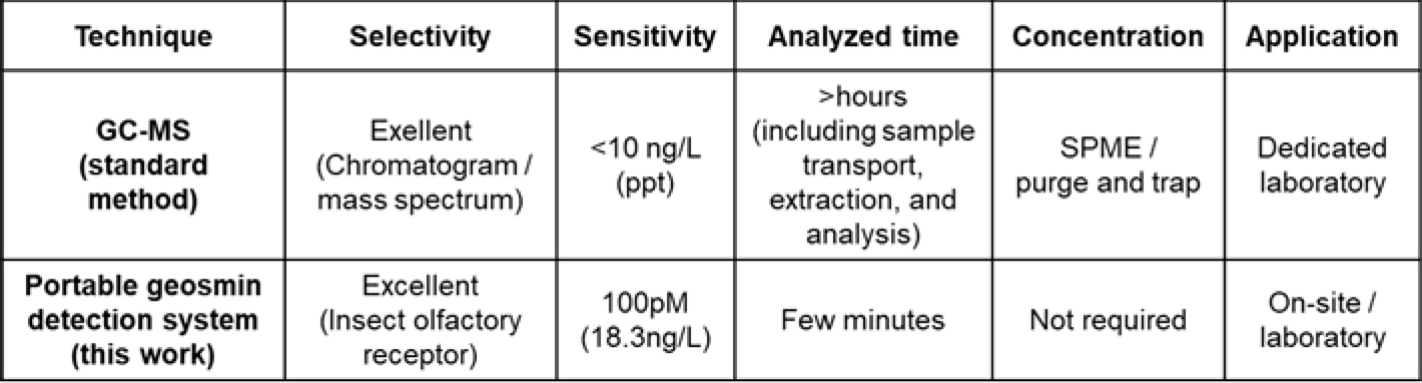
Comparison of the performance of GC-MS and portable geosmin detection system (this work).

Notably, the sensor cell design accommodates integration with a diverse range of insect ORs, as many insect species possess extensive repertoires of olfactory receptors operating via similar mechanisms^27,29–31^. For example, insects are capable of detecting volatiles associated with human perspiration, disease states, and even explosives. The odorant response traits of *D. melanogaster* have been extensively characterized, as recorded in the DoOR database^46,47^. Using such resources, it is feasible to expand this platform to detect a wide array of target odorants by employing alternative ORs. We have also developed a technology to dissolve volatile organic compounds (VOCs) using a fine mist and demonstrated that sensor cells exhibit a fluorescence intensity change in the presence of odorants dissolved from the gas phase^51^. This adaptability enables broad applications, including water quality testing, environmental analysis, biomedical diagnostics, and hazardous substance detection.

Our current detection approach utilizes a Quantus™ fluorometer to measure fluorescence from a single sensor cell type. As the Quantus™ system supports dual-channel detection (green and red fluorescence), it is possible to monitor responses from two distinct sensor cell populations labeled with different reporters. Prior work from our group has also established an array-based chip platform featuring four sensor cell types on a glass substrate, facilitating pattern-based fluorescence analysis^39^. Advancing this concept with a detection system will enable streamlined analysis through multi-cell fluorescence pattern recognition.

The Sf21 cells derived from the fall armyworm (*S. frugiperda*) used in the current study to generate sensor cells support high-level protein expression, particularly for membrane proteins. Alternatively, S2 cells from *D. melanogaster* may offer practical advantages for expressing Drosophila-specific olfactory receptors. Although we successfully established multiple stable expression lines in Sf21 cells (Fig. S1, Table S1), exploring alternative host cell lines could further optimize the efficiency of generating sensor cells.

When measured on-site, samples prepared with surface water displayed lower fluorescence intensity than those prepared with ultrapure water (Fig. 7D), potentially due to background geosmin levels leading to sensor habituation. Environmental samples, such as surface water from the dam reservoir, contain various odorants and minerals that may alter the ionic composition and affect sensor cell response, particularly as the calcium ion concentration directly affects fluorescent output. In the surface water, we observed a decrease in the fluorescence intensity of the sensor cells during the control stimulation without geosmin (Fig. 4B, C). The ionic composition of water fluctuates based on the water collection site and season^53^. However, we found no significant differences in relative fluorescence intensity changes between ultrapure and surface water when proper calibration was applied (Fig. 4C), underscoring the importance of effective calibration methods between matrices (e.g., environmental vs. control samples).

Fluorescence decay of sensory cells, attributable to GCaMP photobleaching^54^ and olfactory receptor inhibitors, was observed under fluorescence microscopy (Fig. 4B). Continuous excitation led to gradual signal decreases, and chemical inhibitors targeting the olfactory receptors and Orco ion channel may also contribute to this attenuation^55^. To mitigate fluorescence intensity decay, future studies should minimize excitation periods and adjust for inhibitor effects to enhance signal stability.

Regarding sensitivity toward geosmin, the detection limit of individual cells under a fluorescence microscope was 100 nM, whereas that of the cell-immobilized cartridge was 100 pM, indicating a marked improvement in sensitivity (Figs. 3F and 6C). This difference is likely attributable to stimulus delivery (continuous exposure in cartridge vs. perfusion during microscopy) and cell immobilization strategies, which allow more cells to contribute to the overall signal in the cartridge. In fluorescence microscopy, fluorescence intensity is measured from individual cells by applying a stimulus for 15 s using a perfusion system. In contrast, in the cartridges, cells are immobilized at the detection site and, in addition to not being perfused, the structure facilitates simultaneous capture of fluorescent signals from multiple cells. Hence, the odorant stimulus is not immediately washed away by perfusion; the cells are constantly exposed to it, and the fluorometer’s photodetector can detect cells that exhibit a fluorescence intensity change above a certain threshold.

Variability in response values—such as the discrepancy between 13.7% (dam lake surface water) and 21.2% (control)—was attributed to trace amounts of geosmin in the assay buffers, potentially reducing the fluorescence response to 100 µM geosmin. This highlights the need for enhanced cartridge preparation and sample pretreatment protocols.

Despite the successful demonstration of on-site detection, several challenges with our platform persist. First, the single-use sensor cell-immobilized cartridge is currently designed to be used immediately after preparation, although viability beyond six days has been observed for Sf21 cells expressing GFP^38^. Hence, improved storage techniques are needed to improve user convenience and extend usability. Second, the current sample introduction process requires pipetting, which may present challenges for non-specialists. Therefore, it is essential to refine both the cartridge and detector to enhance accessibility. By developing a dedicated fluorescence detector for sensor cell responses, we can design a more user-friendly cartridge that simplifies sample introduction and offers an improved measurement method. Third, while the system is now optimized for liquid samples, adapting it for the detection of gaseous odors will demand further advancements in sampling and sensor integration. We previously demonstrated that gas detection is possible using controlled liquid-layer thickness^45^ and fine-bubble entrapment^51^. By incorporating these technologies, the system could be modified to detect gases, expanding its potential applications beyond just liquid solutions.

Overall, we successfully developed a portable, user-friendly geosmin detection system based on insect-derived sensor cells, achieving a detection threshold of 100 pM (18.2 ppt). Combining this approach with available concentration techniques, such as solid-phase extraction^11^, holds promise for comprehensive detection of geosmin contamination in diverse settings, from field sites to household tap water.

## Methods

### Odorants and cells

Geosmin (077–01911; Wako Pure Chemical Industries, Ltd., Osaka, Japan) and 1-octen-3-ol (O5284-25G; Sigma-Aldrich) were dissolved in dimethyl sulfoxide (DMSO; 049–07213; Wako Pure Chemical Industries, Ltd., Osaka, Japan) to prepare the stock solutions (final concentration = 100 mM). The stock solutions were stored at − 80 °C until use. The stock solutions were diluted to the indicated concentrations with assay buffer (140 mM NaCl, 5.6 mM KCl, 4.5 mM CaCl_2_, 11.26 mM MgCl_2_, 11.32 mM MgSO_4_, 9.4 mM D-glucose, and 5 mM HEPES, pH 7.2) to obtain the odorant stimuli.

Sf21 cells (B82101; Thermo Fisher Scientific, Waltham, MA, USA) were incubated and cultured at 27 °C in Grace’s Insect Medium, Supplemented (11605-094; Gibco), containing 10% US Insect Cell Screened fetal bovine serum (FBS) (SH30070.03; GE Healthcare). The cells were cultured so that the cell number increased to 1–3 × 10^6^ cells/mL every 3–4 days. The cells and fresh medium were mixed in a 1:5 ratio, and the cells were passaged repeatedly.

The sensor cell cultures were exposed to three antibiotics diluted in Grace’s Insect Medium: Gentamicin Reagent Solution at a final concentration of 10 µg/mL (prepared from a 10 mg/mL stock solution, 15710-064; Gibco), Blasticidin S HCl at a final concentration of 10 µg/mL (10 mg/mL stock solution, A1113903; Gibco), and Zeocin at a final concentration of 100 µg/mL (100 mg/mL stock solution, R25001; Invitrogen).

### Construction of expression vectors and transfection

Expression vectors for transfecting the OR genes, *Orco*, and *GCaMP6s*, were constructed as previously described^38,39^. WT genes for Or13a (Accession number: NM_078635.3), Or56a (Accession number: NM_079072.2), and Orco (Accession number: AY567998.1) were obtained from *D. melanogaster* antennal cDNA, and codon-converted *Or56a* was obtained by gene synthesis (Integrated DNA Technologies, Inc., IA, USA). For the codon conversion, 296 of the 1,260 base pairs (23.5% conversion of total) were converted according to the codon usage of the host, *Spodoptera frugiperda*, using the codon usage database of Kazusa DNA Research Institute (https://www.kazusa.or.jp/codon/). The base sequence of codon-converted *Or56a* was converted to match the amino acid sequence of the WT Or56a protein according to the codon usage database of Kazusa DNA Research Institute.

A dual expression vector containing the target OR and *Orco* gene was generated. First, *Orco* was amplified through PCR with PrimeSTAR (R010A; TAKARA BIO INC.) using the following primers for the *Orco* gene and inserted into the HindIII and SacII sites in the Multi Cloning Site (MCS) of the pIZ/V5-His vector (V8000-01; Thermo Fisher Scientific) with the NEBuilder HiFi DNA Assembly Master Mix (E2621; NEB). *Orco* forward: 5′-TTCGAATTTAAAGCTGCCGCCATGATGACAACCTCGATGCAGCC-3′; *Orco* reverse: 5′-TTACCTTCGAACCGCTTACTTGAGCTGCACCAGCAC-3′.

Next, the expression cassette from the promoter to the poly (A) signal, including the *Orco* gene, in the constructed vector was PCR-amplified with PrimeSTAR and inserted into the Pci1 site of the pIB/V5-His vector (V8020-01; Thermo Fisher Scientific). The target OR gene was then inserted into the EcoR1 and Xho1 sites of the MCS of the Orco-incorporated pIB vector with the NEBuilder HiFi DNA Assembly Master Mix. Finally, an expression vector was constructed by inserting the *GCaMP6s* gene^56^ into the MCS of the pIZ/V5-His vector.

Transfection into Sf21 cells was performed using the TransIT-Insect Transfection reagent according to the manufacturer’s instructions (MIR6100; Mirus Bio LLC, Madison, WI, USA). Approximately 48 to 72 h after transfecting Sf21 cells with the dual expression pIB vector carrying the OR and *Orco* genes, as well as the pIZ vector expressing the *GCaMP6s* gene, the cells were cultured in Grace’s Insect Medium with three antibiotics: Blasticidin S HCl, Zeocin, and Gentamicin. These transfected cells were then used for calcium imaging or single-cell isolation.

### Extraction of single cells and generation of cell lines

Single cells were isolated using the limiting dilution method. First, Sf21 cells co-transfected with the pIB/V5-His vector containing the target insect OR and *Orco* genes and pIZ/V5-His vector containing the *GCaMP6s* gene were transferred into T-25 cell culture flask (353082; Corning, NY, USA) using 6 mL of medium containing the three antibiotics: Gentamicin, Blasticidin S HCl, and Zeocin. Upon reaching confluence, 6 mL of cell culture supernatant was collected from the flasks, placed in 15-mL tubes (91015; TPP, Switzerland), and centrifuged at 400 ×*g* for 3 min at 4 °C. After centrifugation, the supernatant medium was sterilized using a 0.45-µm filter (431220; Corning) and then mixed with an equal volume of fresh medium containing antibiotics (Blasticidin S HCl at a final concentration of 10 µg/mL and Zeocin at a final concentration of 100 µg/mL) to prepare the conditioned medium.

Cells attached to the bottom of the flask from which the supernatant was collected were suspended in 1 mL of fresh Grace’s Insect Medium, and the cells were counted. Next, the cell suspension containing approximately 40 cells was added to the conditioned medium, and 100 µL aliquots were added to each well of a 96-well plate (3860-096; IWAKI).

After confirming that each well contained only a single cell using an inverted microscope (IX73; Olympus, Tokyo, Japan), the plate was incubated at 27 °C until 80%–90% confluency. The culture was then scaled up in the following order: 24-well plate (3820-024; IWAKI), 35 mm dish (353801; Corning), and T-25 cell culture flask. Cells that could be scaled up to T-25 flasks were examined for a fluorescence response via calcium imaging and grouped according to the amount of change in the fluorescence intensity (Excellent: ≥10%; Good: 5%–10%; Poor: 1%–5%). Cell lines with excellent and good responses were considered homogeneous cell clones.

Numerous cells were required to prepare the cell-immobilized cartridge; therefore, the cells were cultured in Erlenmeyer flasks (4113-0500; Thermo Fisher Scientific). Flasks containing 120 mL of the cell suspension were placed in a shaking incubator (FMC-100; TOKYO RIKAKIKAI CO., LTD., Tokyo, Japan) or multi shaker (MMS-310; TOKYO RIKAKIKAI CO., LTD.) set at 60 rpm and 27 °C. For cell culture, 80 mL of culture medium in each flask was replaced with the same volume of fresh medium every 4 days.

Gene expression in the sensor cells was confirmed using reverse transcription (RT)-PCR. Cells were collected from each cell culture flask (5 mL) and suspended in 500 µL of TRIzol™ Reagent (15596026; Invitrogen). Total RNA was extracted according to the manufacturer’s instructions. Genomic DNA was removed by Recombinant DNase I (2270A; TAKARA BIO INC, Shiga, Japan), and the concentration of total RNA was measured. cDNA was synthesized from 1 µg of total RNA using the RNA PCR Kit (AMV) Ver.3.0 (RR019A; TAKARA BIO INC). PCR was performed using ExTaq polymerase (RR006A; TAKARA BIO INC) and cDNA as the template for 30 cycles. The following primers were used: *Or56a* forward, 5′-ATGTTTAAGGTTAAGGACTTGCTGCTGTCTC-3′, *Or56a* reverse, 5′-TTAGTAGAGGTGGCTACTACGGAGG- 3′; *Or13a* forward, 5′-ATGTTCTATTCGTATCCCTACAAAGCAC-3′, *Or13a* reverse, 5′-TTAATCTAGTTTCTTTTCGTCGTCGAAG-3′; *Orco* forward, 5′-ATGATGACAACCTCGATGCAGCC-3′, *Orco* reverse, 5′-TTACTTGAGCTGCACCAGCAC-3′; *GCaMP6s* forward, 5′-ATGGGTTCTCATCATCATCATC-3′, *GCaMP6s* reverse, 5′-TCACTTCGCTGTCATCATTTGTAC-3′.

### Calcium imaging

The fluorescence response of cells was determined using a perfusion measurement chamber. Cells were seeded onto a 12 mm-diameter cover glass (CS-12R; Warner Instruments, LLC, Hamden, CT, USA) and allowed to adhere, then placed in a Quick Change Chamber (RC-48LP; Warner Instruments). Silicon tubes (1 mm inner diameter) connected to two peristaltic tube pumps (MP-2010; TOKYO RIKAKIKAI CO., LTD.) were connected to the inlet and outlet of the Quick Change Chamber using tube clamps (CAT-1; NARISHIGE Co., Ltd., Tokyo, Japan) and perfused with the assay buffer solution. The stimulus solution was introduced into the chamber by switching the inlet tube end from the assay buffer bottle to the stimulus solution bottle. The chamber volume was set to 230 µL, the flow rate to 1.4 mL/min, and the stimulus solution was supplied for 15 s.

Cell fluorescence intensity changes were measured using an upright fluorescence microscope (BX51WI; Olympus) equipped with a 20× water immersion objective (UMPlanFI 20×/0.50 W; Olympus) and an EM-CCD camera (DU-897E: Andor Technology PLC, Belfast, UK). The fluorescence filter was a mirror unit for green fluorescence protein (GFP) (U-MGFPHQ; Olympus), and the light source was a 100 W halogen lamp (TH4-100; Olympus). Fluorescence images of cells were acquired using an Andor iQ 3.2 (Andor Technology PLC, Belfast, UK), with an exposure time of 500 ms and a frame rate of 1 frame per second.

The acquired fluorescence images were analyzed using MATLAB R2024b (Mathworks, Natick, MA, USA). A region of interest was set to the region of appropriate brightness using the circular Hough transform^57,58^, and the cell position was selected. The extent of change in fluorescence intensity (ΔF/F_0_) was calculated using Eq. (1):

1 $$\Delta\:F/F_0\:=\:(F_t-F_0)/F_0\:\times\:\:100$$

where F_t_ is the fluorescence intensity at time t, and F_0_ is the fluorescence intensity before the arrival of the stimulus (average value for 30 s).

In the evaluation of odorant selectivity and dose responsiveness, the fluorescence intensity change in response to each stimulus solution was calculated by subtracting the average 20 s fluorescence intensity change at the peak response from the average 20 s fluorescence intensity change just before the arrival of the stimulus solution. To evaluate the selected cell lines, the fluorescence intensity of all images was calculated using Andor iQ, and the fluorescence intensity across the entire screen was derived from these calculations to determine the change in fluorescence intensity. In addition, the difference between the lowest fluorescence intensity from the time after the stimulus substance influx to the time of peak response, and the fluorescence intensity at a single point within the peak, was used as the cell response change value.

### Preparation of the sensor cell-immobilized cartridges

The cell-immobilization cartridge consists of a single glass fragment placed inside each 0.5 mL PCR tube (E4941; Promega). To prepare glass pieces for cell immobilization, 76 mm × 26 mm glass slides (S9441; Matsunami Glass Ind., Ltd., Osaka, Japan) were cut into approximately 4 mm × 26 mm pieces using a glass cutter (419–9847; TRUSCO NAKAYAMA Corp., Tokyo, Japan). Alternatively, we purchased custom-made glass fragments (3.4 mm × 26 mm × 1 mm; Matsunami Glass Ind., Ltd.) and treated the entire surface with reagents to immobilize the cells. Approximately 100 µL of collagen solution was dropped onto the glass pieces, which were then placed on a clean bench for 20 min. The collagen solution was prepared by diluting Cellmatrix Type I-C (631-00771; Nitta Gelatin Inc., Osaka, Japan) 10 times with HCl (pH 3). After removal of excess collagen solution from the glass surface, 100 µM of the cell-immobilization agent (oleyl-PEG-NHS) was added, and the glass was incubated at 37 °C for more than 20 min. For the cell immobilization agent, 0.4 mg of SUNBRIGHT (OE-040CS; NOF corporation, Tokyo, Japan) was mixed with 10 µL of super-dehydrated DMSO (048-32811; FUJIFILM Wako Pure Chemicals Co.) and diluted 100 times with D-PBS(-) (045-29795; FUJIFILM Wako Pure Chemicals Co.). The glass was then washed with ultrapure water and air-dried to obtain the cell-immobilization-agent-treated glass slide.

Next, sensor cells were collected by centrifugation and suspended in the assay buffer solution (140 mM NaCl, 5.6 mM KCl, 4.5 mM CaCl_2_, 11.26 mM MgCl_2_, 11.32 mM MgSO_4_, 9.4 mM D-glucose, and 5 mM HEPES, pH 7.2) to obtain a final count of 1 × 10^7^ cells/mL. The cell suspension was dropped onto the glass slide coated with the cell immobilization agent and incubated at room temperature (~ 25 °C) for 10 min. Unattached cells were washed off using an assay buffer to obtain the sensor cell-immobilized glass slide. The cell-immobilized cartridges were prepared by setting them inside a 0.5 mL PCR tube containing 400 µL of assay buffer with a final concentration of 0.1% DMSO.

### Simple measurement using a sensor cell-immobilized cartridge

Fluorescence from the sensor cell-immobilized cartridge was measured using the Quantus™ Fluorometer (E6150; Promega Corporation, Madison, WI, USA). The Quantus™ Fluorometer is a highly sensitive compact fluorometer for nucleic acid quantification equipped with two detection systems: green fluorescence (Ex. 495 nm/Em. 510–580 nm) and red fluorescence (Ex. 640 nm/Em. 660–720 nm). This fluorometer can be connected to a PC with a USB cable, and the acquired data can be displayed and saved using the dedicated Quantus™ Software (Promega Corporation). First, the sensor cell-immobilized cartridge was placed in the Quantus™ Fluorometer, and a calibration step was performed in which the fluorescence intensity was repeatedly acquired until more than 5 values were obtained, each with a change in fluorescence intensity within ± 2%. Then, 200 µL of the sample solution was gently added and mixed by pipetting, and fluorescence intensity was measured every 20 s for 140 s. The odorant stimuli and the samples were diluted with the assay buffer to contain 0.1% DMSO. The acquired data with Quantus™ Software were analyzed using Microsoft Excel (Microsoft Corporation, Redmond, WA, USA). The mean value for the 10 points before stimulation was taken as F_0_, and the rate of change of fluorescence intensity was calculated using Eq. (2):

2 $$\:\varDelta\:F/F_0\:\left(Quantus\right)\:\left(\mathrm{\%}\right)\:=\:(F_j\:-\:F_0)/F_0\:\times\:\:100$$

where F_j_ is the fluorescence intensity of the acquisition after the stimulation.

The change in fluorescence intensity of the sensor cell-immobilized cartridge was calculated using ∆F⁄F_0_ (Quantus) (%) as an evaluation index, and the response value 120 s after sample addition was evaluated.

### Preparation of samples for on-site detection

For on-site detection, environmental samples were prepared using the surface water collected at the lakeside of the Reservoir. On the sampling day, the temperature at the lakeside was approximately 26.0 °C, and the relative humidity was 68%. The water, collected into 50 mL tubes (91050; TPP), was filtered (Millex, Millipore) to remove suspended materials (microorganisms, etc.). Next, the filtered surface water was mixed with 5× assay buffer to prepare 1× surface water assay buffer, which was used to prepare sensor cell-immobilized cartridges and for on-site measurement. Surface water buffer solution was used as the control, and a buffer solution containing 100 µM geosmin was used as the test sample. Thus, the only difference was whether the water used in the tests was surface water from the reservoir or ultrapure water.

### Statistical analysis

Welch’s *t*-test was used to compare sample pairs. For data on odorant selectivity in the sensor cells and the cell-immobilized cartridges, data from two groups (control and target odorant) were tested. For dose-dependent response data, differences between the control and each concentration of the target odorant were tested. The *p*-values were adjusted using the Holm–Bonferroni method for multiple comparisons, and significance levels of 5% (*), 1% (**), and 0.1% (***) were used. Calculations were performed in MATLAB R2024b (Mathworks, Natick, MA, USA). The sample sizes used to test the selectivity and sensitivity of the sensor cells and the cell-immobilized cartridges are indicated in the captions of each figure.

## Supplementary Information

Below is the link to the electronic supplementary material.

Supplementary Material 1

Supplementary Material 2

## Data Availability

The calcium imaging datasets that were obtained in this study are available from the corresponding authors on reasonable request.
