# Supplementary material for: Portable geosmin detection system based on sensor cells expressing insect odorant receptors
Source: Sci Rep. 2026 Apr 28;16:12577. doi: 10.1038/s41598-026-41786-8 (PMC13125297; doi:10.1038/s41598-026-41786-8)

**Supplementary information**

**Portable geosmin detection system based on sensor cells expressing insect odorant receptors**

Hidefumi Mitsuno *^1, 8^, Shogo Araki ^2, 8^, Yuji Sukekawa ^1, 8^, Daigo Terutsuki ^1, 6, 8^, Sawako Niki ^1^, Eri Kuroda ^1^, Shunsuke Fujibayashi ^2^, Takeshi Sakurai ^3^, Kumiko Oguma ^1, 4^, Satoshi Yamaguchi ^1, 7^, Shinya Yamahira ^5, 7^, Teruyuki Nagamune ^5^, Ryohei Kanzaki *^1, 2^

*Corresponding author. Email: mitsuno@brain.imi.i.u-tokyo.ac.jp; kanzaki@rcast.u-tokyo.ac.jp

**This file includes:**

Supporting text

Figures S1 to S6

Tables S1 to S5

**Other supporting materials for this manuscript include the following:**

Movie S1


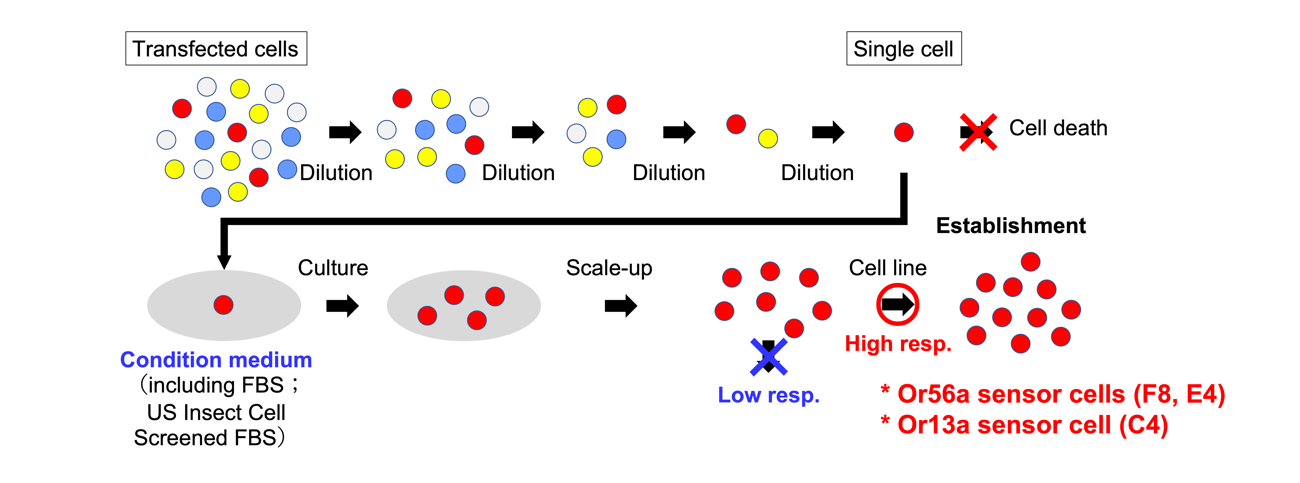


**Figure S1. Schematic illustration of cell clone establishment.**

The Sf21 cell population transfected with odorant receptor and *Orco* and *GCaMP* genes includes cells with strong, weak, or no fluorescence response to the target odorant. To establish sensor cells with strong and homogeneous fluorescence responses, single clones were isolated by repeatedly diluting the transfected cell population. Cell lines with the same genetic background were established by repeatedly passaging the isolated single cells in conditioned medium and increasing the culture scale. Finally, by evaluating the cell lines' responsiveness to target odorants, sensor cells with excellent response characteristics (fluorescence responses >20% across multiple tests) were selected.


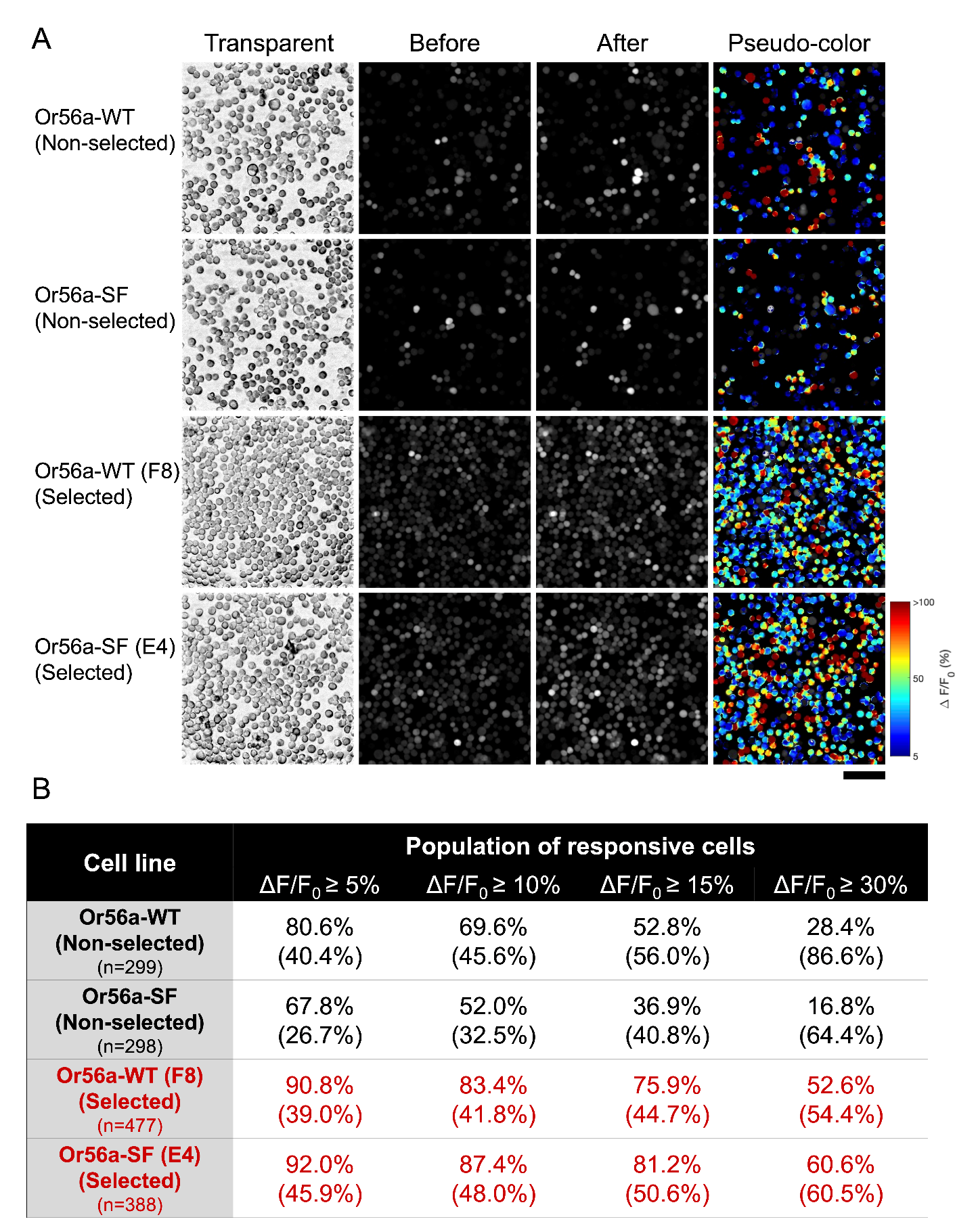


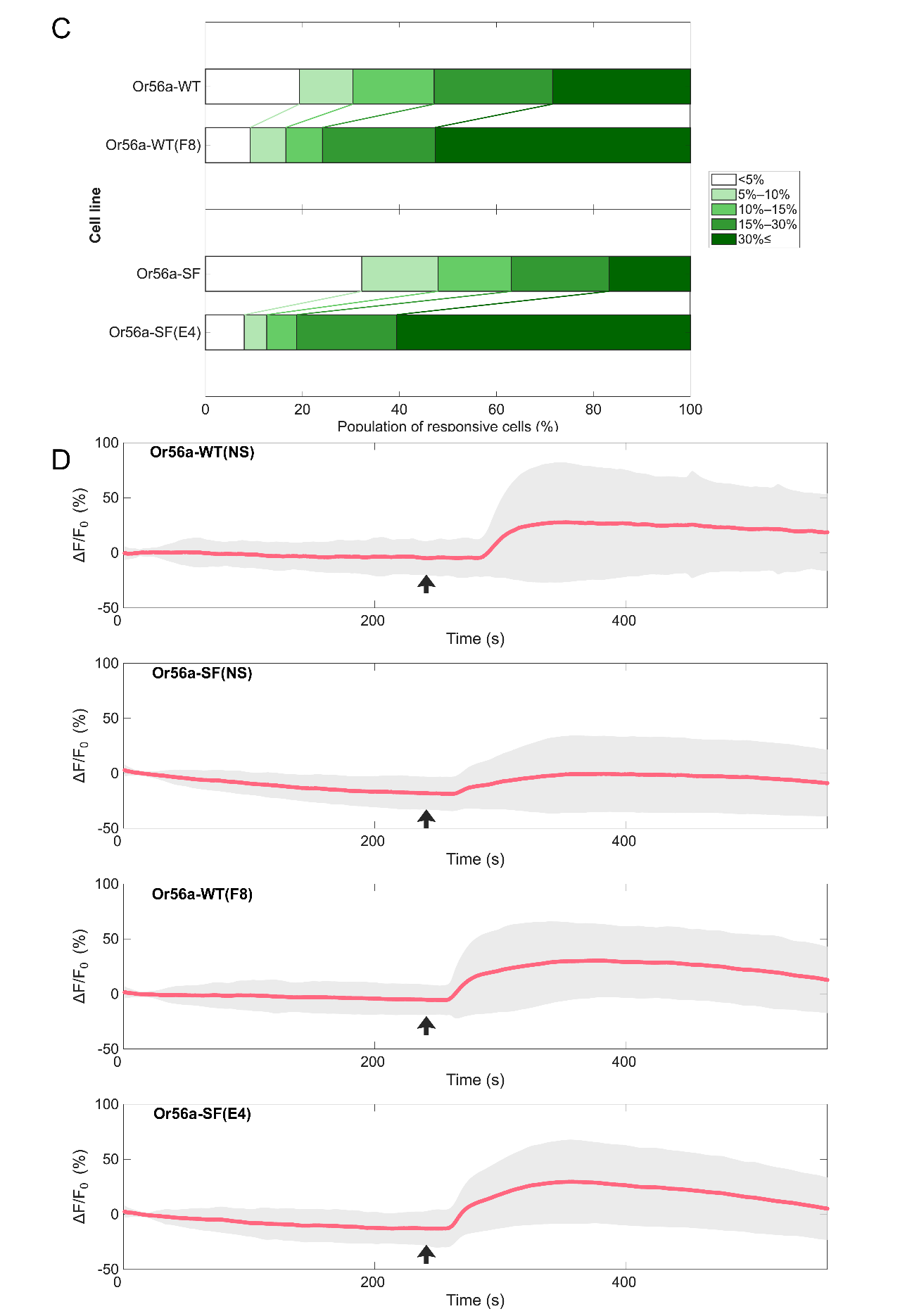


**Figure S2.** **Population of responsive cells and time course changes of fluorescence intensity in non-selected and selected cell lines (Or56a).**

**(A)** Microscope images of typical fluorescence changes in a group of non-selected cells transfected with the Or56a gene and Or56a sensor cells. Listed in order from top to bottom: Or56a-WT (non-selected), Or56a-SF (non-selected), Or56a-WT (F8) (selected), and Or56a-SF (E4) (selected). The left sides show bright-field images; the middle shows fluorescence microscopy images before and after geosmin addition; and the right sides show pseudo-color images of fluorescence responses to geosmin. The non-selected group (upper rows) contains many cells with low fluorescence intensity change, while almost all sensor cells (lower rows) exhibit fluorescence intensity change. The images show pseudo-colors, with dark red indicating greater changes in fluorescence intensity and dark blue indicating smaller changes. Scale bar = 100 µm. **(B)** Percentage of responsive cells in the non-selected cells and sensor cells (F8 and E4). The population of cells exhibiting fluorescence changes (ΔF/F0) of 5%, 10%, 15%, or 30% was counted. The number in parentheses indicates the average change in fluorescence intensity across the cell population. **(C)** Population of responsive cells in either non-selected cells (NS) or sensor cells (F8 and E4) exhibiting fluorescence changes of < 5%, 5%–10%, 10%–15%, 15%–30%, and ≥ 30%. The number of cells exhibiting ≥ 30% change in fluorescence intensity was higher in the selected sensor cells than in the NS cells. **(D)** Time course changes of average fluorescence intensity in the NS and sensor cells (F8 and E4). Or56a-WT(NS); n = 299 cells, Or56a-SF(NS); n = 298 cells, Or56a-WT(F8); n = 477 cells, and Or56a-SF(E4); n = 388 cells. Black arrows indicate the time when the geosmin stimulus (10 µM) was applied. Error ranges (± SD) are shown in gray.


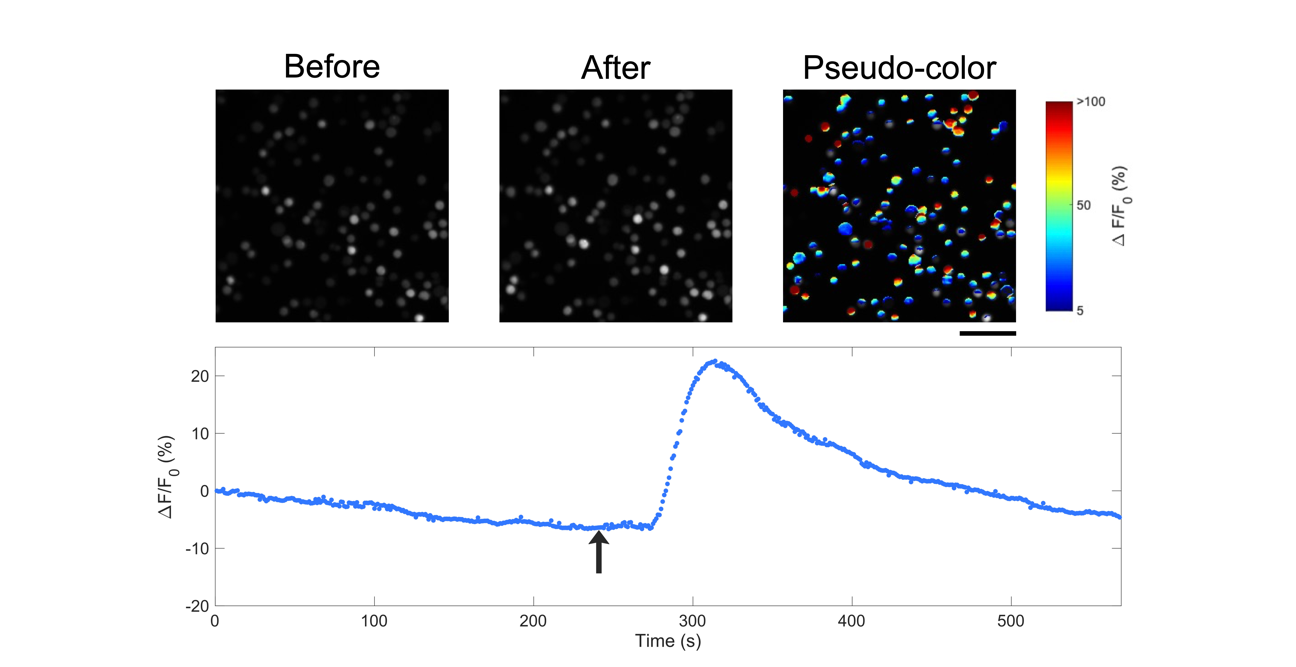


**Figure S3.** **Microscope images and time course changes of fluorescence intensity in the selected cell line (Or13a-WT [C4]).**

Microscope images (upper panels) and time course changes of fluorescence intensity (lower panel) in Or13a-WT (C4). (left) Fluorescence microscopic images before and after 1-octen-3-ol addition; (right) pseudo-color image, with dark red indicating greater changes in fluorescence intensity and dark blue indicating smaller changes. Scale bar = 100 µm. Black arrows indicate the time when the 1-octen-3-ol stimulus (10 µM) was added.


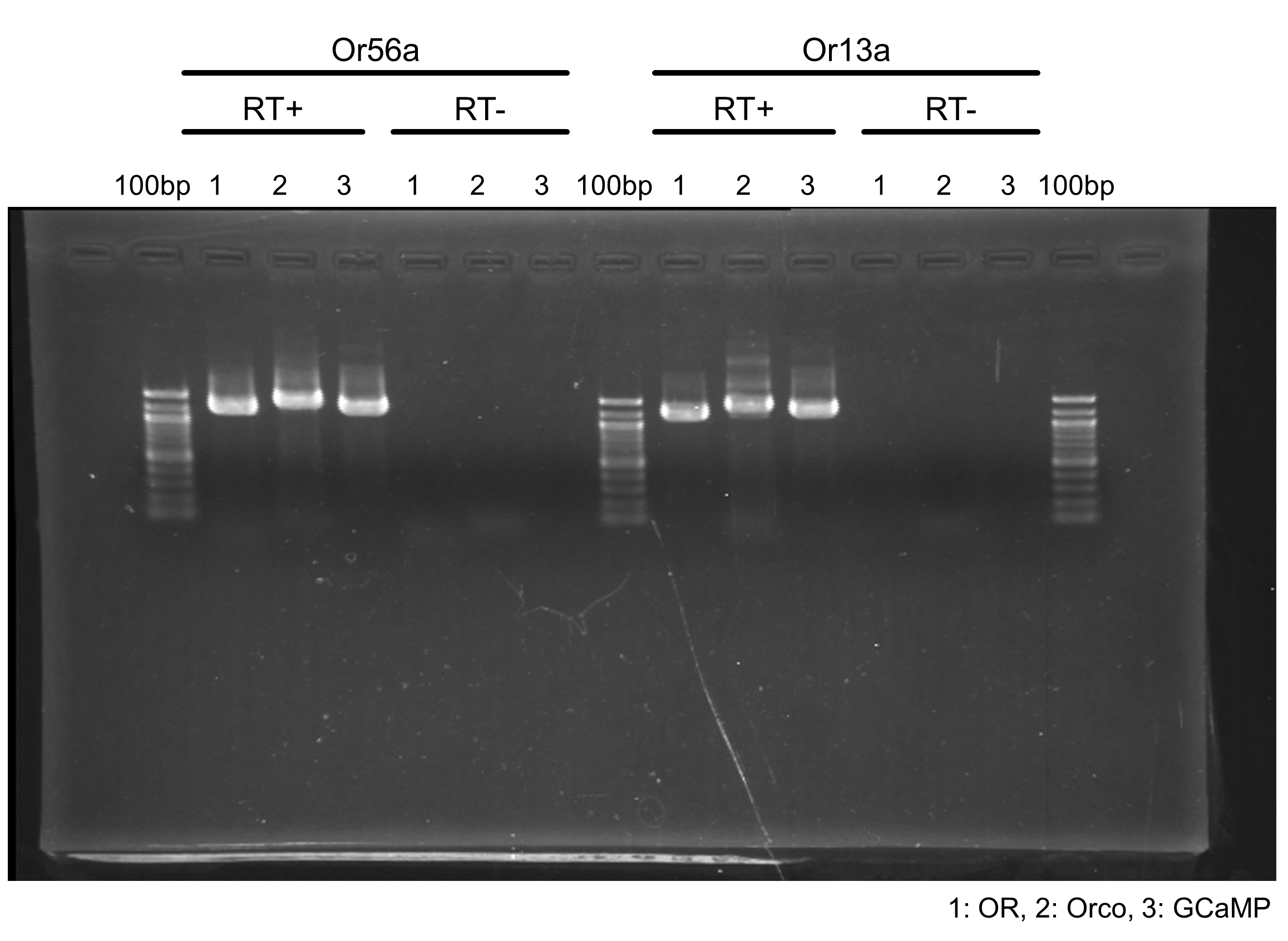


**Figure S4. Gene expression in Or56a-SF-E4 and Or13a-WT-C4 sensor cells.**

Left side shows RT-PCR results in Or56a-SF-E4 sensor cells, and the right side shows the results of Or13a-WT-C4 sensor cells. ‘RT-’ indicates no DNase I treatment, and ‘RT+’ indicates DNase I treatment. Lane 1 represents the odorant receptor (OR), lane 2 Orco, and lane 3 GCaMP6s. 100 base-pair (bp) markers were electrophoresed at both ends and in the center.


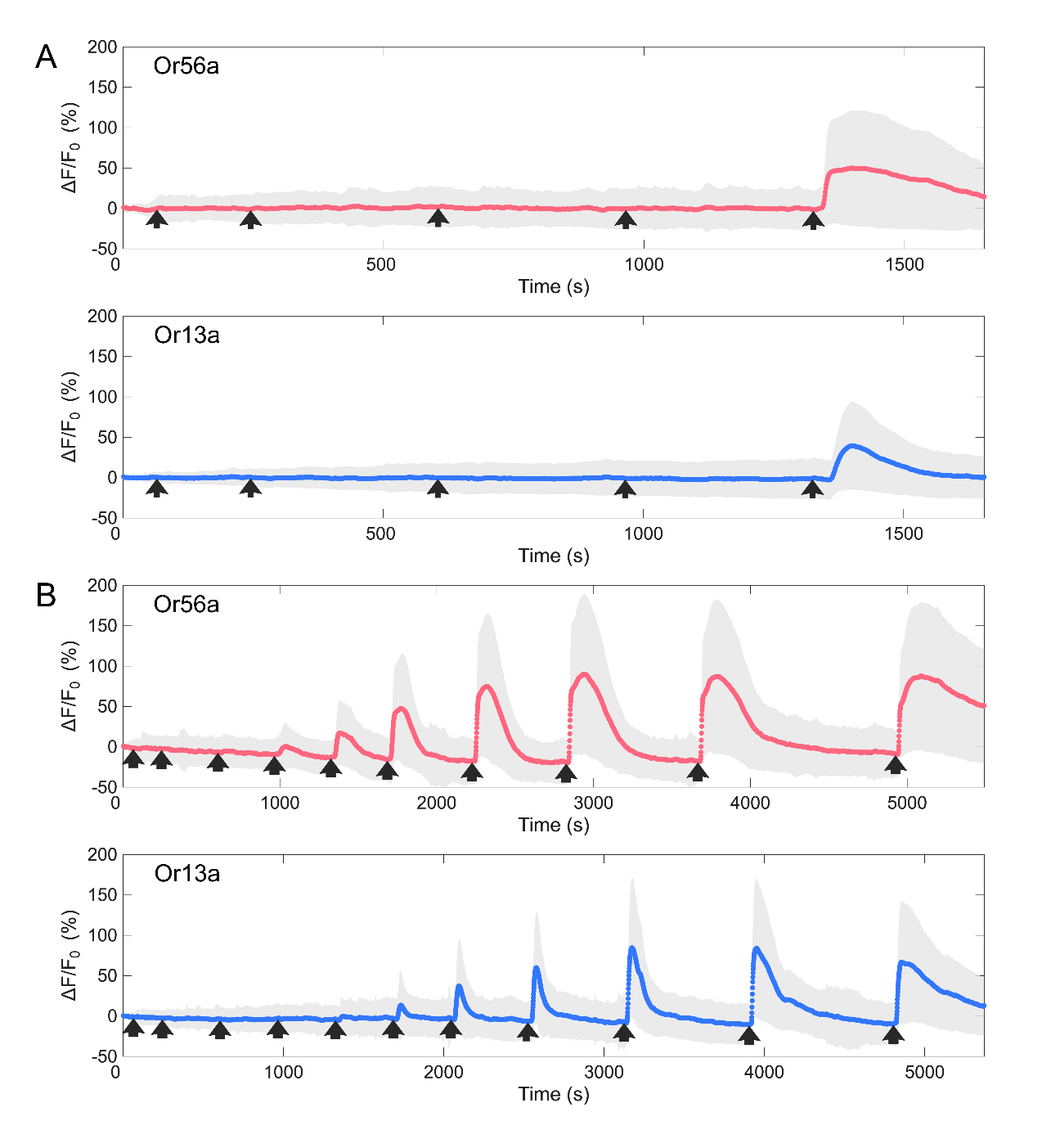
Figure S5. Time course changes in fluorescence intensity to various moldy odorants (selectivity test) and concentrations (sensitivity test) in Or56a sensor cells and Or13a sensor cells.

**(A)** For Or56a sensor cells, a negative control, 2-MIB, 2,4,6-trichloroanisole, 1-octen-3-ol, and geosmin were tested sequentially. For Or13a sensor cells, a negative control, 2,4,6-trichloroanisole, geosmin, 2-MIB, and 1-octen-3-ol were tested. Upward arrows indicate the time when the odorant stimulus was applied. The average values of fluorescence intensity are shown: Or56a, *n* = 127 cells; Or13a, *n* = 199 cells. Error ranges (± SD) are shown in gray. **(B)** Time-course changes in fluorescence intensity at various concentrations of the target odorant. Upward arrows indicate the time when the odorant stimulus was applied. For Or56a sensor cells and Or13a sensor cells, geosmin and 1-octen-3-ol, respectively, were tested sequentially at the following concentrations: negative control, 10 nM, 30 nM, 100 nM, 300 nM, 1 µM, 3 µM, 10 µM, 30 µM, and 100 µM for geosmin, and up to 300 µM for 1-octen-3-ol. The average values of fluorescence intensity are shown; Or56a, n = 230 cells; Or13a, n = 339 cells. Error ranges (± SD) are shown in gray.


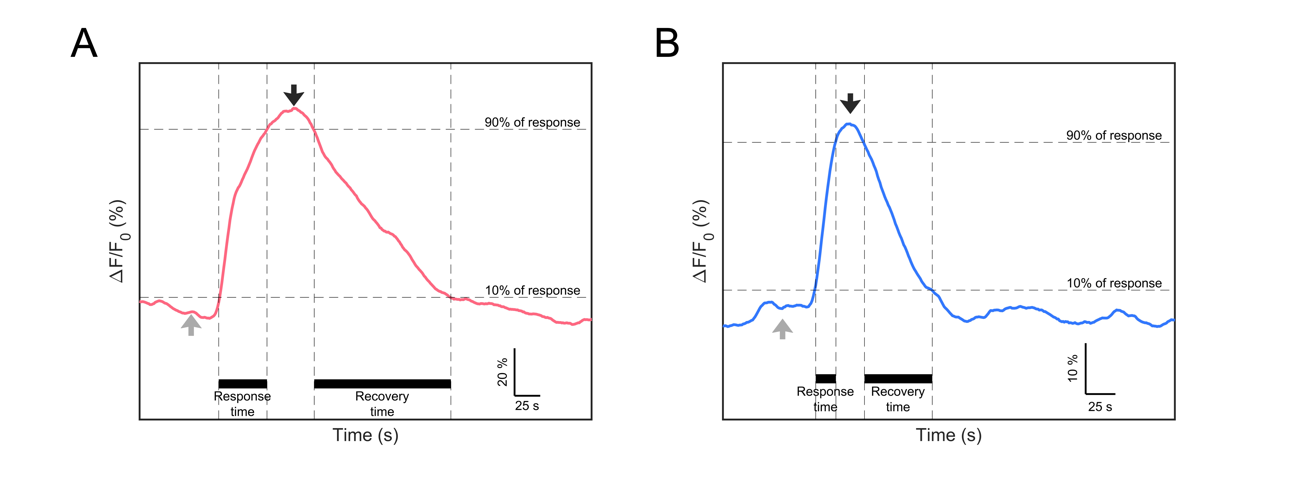


**Figure S6.** **Typical fluorescence response of a cell in sensor cells.**

**(A)** Time course of typical fluorescence intensity change in the Or56a sensor cell. Gray arrow indicates the baseline time just before the geosmin addition. Response time = 47 s; recovery time = 133s. **(B)** Fluorescence intensity in the Or13a sensor cells. Gray arrow indicates the baseline time before 1-octen-3-ol addition. Response time =17 s; recovery time = 57 s.

**Table S1. Number of isolated cells and established sensor cells.**

|  | Single cells | Cell lines (Total) | | | Higher response^#^ |
| --- | --- | --- | --- | --- | --- |
|  |  | < 5 % | 5-15 % | 15 % < |  |
| **Or56a-WT** | 26 | 11 | | | 4 (F8, G2, E3, D9) |
|  |  | 6 | 1 | 4 |  |
| **Or56a-SF** | 35 | 19 | | | 4 (E4, E11, C2, C3) |
|  |  | 10 | 5 | 4 |  |
| **Or13a-WT** | 14 | 4 | | | 1 (C4) |

#:Alpha-numeric labels (e.g., F8 or E4) are derived from the coordinate of the well in the 96-well plate where the single cells were cultured.

**Table S2. Fluorescence intensity changes exhibited by each cell line.**

| **Codon** | **Name of cell lines** | **Fluorescent int. (%)** | **Evaluation^#^** |
| --- | --- | --- | --- |
| WT | C6 | 0.5 | - |
|  | C7 | ND | - |
|  |  | ND | - |
|  | C8 | 0.7 | - |
|  | D9 | 14.5 | Good |
|  |  | 15.1 | Excellent |
|  | E3 | 18 | Excellent |
|  |  | 19.8 | Excellent |
|  | E7 | 4.7 | Poor |
|  | E12 | 0.9 | - |
|  |  | ND | - |
|  | F3 | ND | - |
|  | **F8** | **31** | **Excellent** |
|  |  | **20** | **Excellent** |
|  | G2 | 13.8 | Good |
|  |  | 25.6 | Excellent |
|  | G8 | 7.5 | Good |
| SF | B1 | ND | - |
|  |  | ND | - |
|  | B2 | 4.9 | Poor |
|  |  | 6.4 | Good |
|  | B5 | ND | - |
|  |  | ND | - |
|  | C2 | 21 | Excellent |
|  |  | 10 | Good |
|  | C3 | 15.2 | Excellent |
|  |  | 8.6 | Good |
|  | C5 | 0.6 | - |
|  |  | 1.7 | Poor |
|  | C6 | 7 | Good |
|  |  | 6.2 | Good |
|  | C12 | 3.9 | Poor |
|  |  | 2.6 | Poor |
|  | D1 | 4.3 | Poor |
|  |  | 3.8 | Poor |
|  | **E4** | **35.7** | **Excellent** |
|  |  | **30.3** | **Excellent** |
|  |  | **24** | **Excellent** |
|  | E6 | 2.8 | Poor |
|  |  | 1 | Poor |
|  | E8 | ND | - |
|  | E11 | 38 | Excellent |
|  |  | 16 | Excellent |
|  |  | 5.6 | Good |
|  |  | 3.8 | Poor |
|  | F6 | 5.4 | Good |
|  |  | 5.1 | Good |
|  | G5 | 1 | Poor |
|  |  | 1.8 | Poor |
|  | G6 | ND | - |
|  | G7 | ND | - |
|  |  | ND | - |
|  | G11 | 5.2 | Good |
|  |  | 12.1 | Good |
|  |  | 5.7 | Good |
|  | H5 | 12.5 | Good |

#: Excellent: ≥ 10%; Good: 5–10%; Poor: 1–5%.

The selected sensor cells are shown in bold.

**Table S3. Fluorescence intensity changes exhibited by each cell line.**

| Codon | Name of cell lines | Fluorescent int. (%) | Evaluation^#^ |
| --- | --- | --- | --- |
| WT | **C4** | **16.7** | **Excellent** |
|  |  | **10.7** | **Good** |
|  |  | **8.1** | **Good** |
|  | D8 | 10.6 | Good |
|  |  | 5.8 | Good |
|  | F2 | 6.7 | Good |
|  |  | 5.9 | Good |
|  | G9 | 2.2 | Poor |

#: Excellent: ≥10%; Good: 5–10%; Poor: 1–5%.

The selected sensor cells are shown in bold.

**Table S4. Concentration of moldy odor (geosmin and 2-MIB) in the lakeside-collected surface water based on GC/MS analysis.**

|  | **Geosmin** | **Conc. (Geo)** | **2-MIB** | **Conc.**  **(2-MIB)** |
| --- | --- | --- | --- | --- |
| **Total** | 0.055 µg/L | 301.7 pM | 0.012 µg/L | 71.3 pM |
| **Outside of Algae** | 0.019 µg/L | 104.2 pM | 0.010 µg/L | 59.4 pM |

"Total" represents the sum of the amount stored inside the algae and the amount outside the algae (Outside of Algae). GC/MS analyses were performed under the conditions reported in Table S5.

**Table S5. Analysis conditions for GC/MS with purge and trap concentration for the dam lake water samples.**

| **System** | |  |
| --- | --- | --- |
|  | Purge and trap | PT6000 (GL Science, Tokyo, Japan) |
|  | GC/MS | GCMS-QP2020 (Shimadzu, Kyoto, Japan) |
|  | Column | Inert Cap 5MS/Sil  (0.25 mm I.D. × 30 m, df = 0.50 µm, GL Science, Tokyo, Japan) |
|  |  |  |
| **PT condition** | |  |
|  | Purge time | 12 min |
|  | Purge flowrate | 60 mL/min |
|  | Sample heater | 60 °C |
|  | Dry purge time | 1 min |
|  | Desorb temperature | 220 °C |
|  | Desorb time | 3 min |
|  | Trap | AQUATrap-1 |
|  | Sample volume | 20 mL |
|  |  |  |
| **GC condition** | |  |
|  | Column temperature | 60 °C (1 min hold) - 4 °C/min - 120 °C - 10 °C/min - 170 °C - 20 °C/min - 220 °C (5 min hold) |
|  | Injection | Split 1:5, 150 °C |
|  | Purge flowrate | 2.5 mL/min |
|  | Carrier gas | He, 120 kPa |
|  |  |  |
| **MS condition** | |  |
|  | Interface temperature | 250 °C |
|  | Ion source temperature | 200 °C |
|  | Measurement mode | Selected ion monitoring (SIM) |
|  | Event time | 0.3 s |
|  | Monitoring ion | 95, 107, 108, 111, 112, 125, 213, and 215 m/z |
|  |  |  |

**Movie S1.**

**A series of the operations in measurement using a sensor cell-immobilized cartridge.**

A sensor cell-immobilized cartridge was set on the fluorometer, the baseline fluorescence was measured, and then the fluorescence after the sample addition was acquired. The acquired fluorescence intensity was then analyzed to calculate the amount of change in fluorescence intensity after the sample addition. The elapsed time of the series of these operations is shown in the lower right corner.


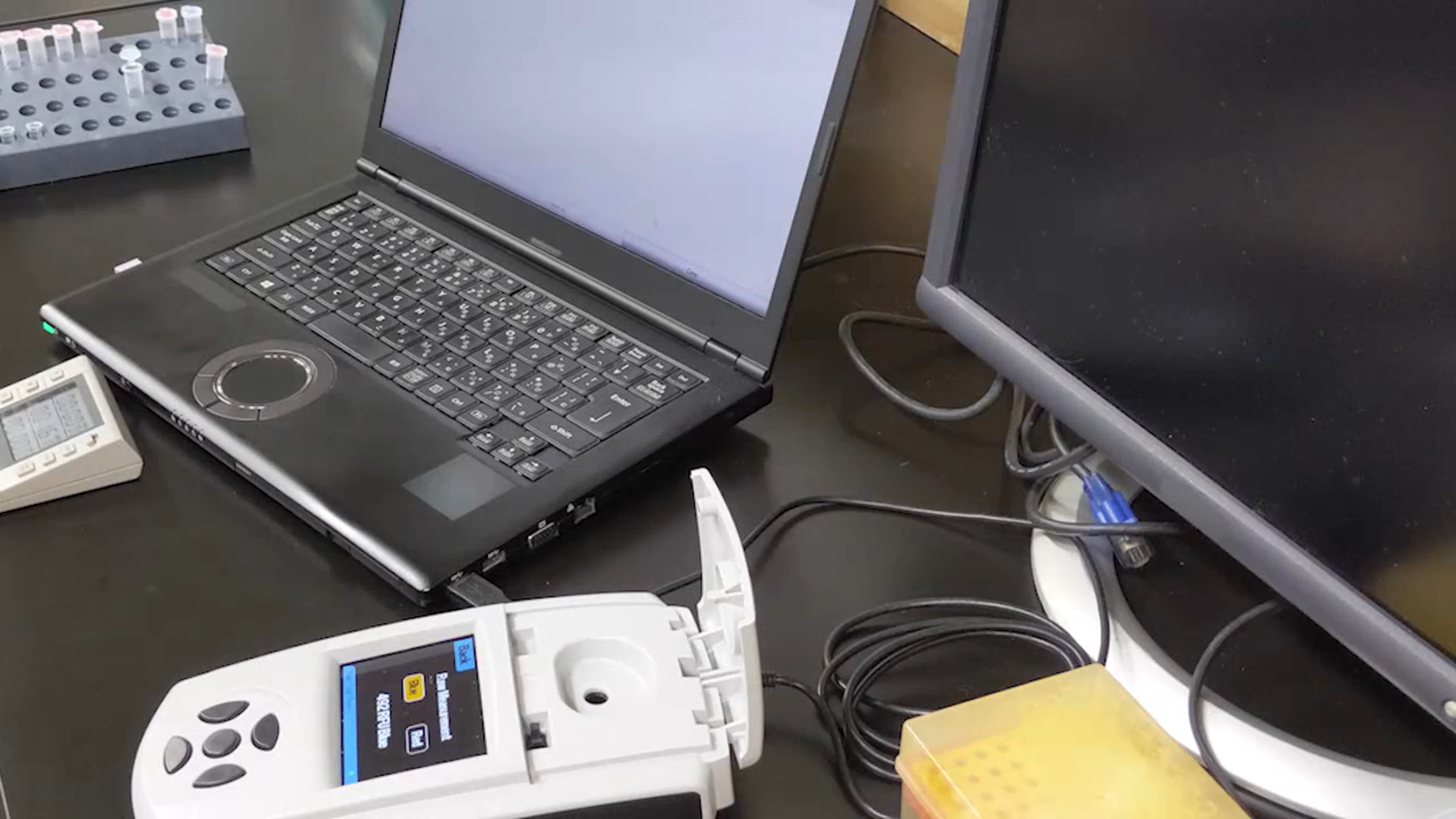

Supplement: Supplementary file 1 — Supplementary Material 1 [file 41598_2026_41786_MOESM1_ESM.docx]
